# The *Moniliophthora roreri* Effector *Mr* B1 Affects Cellular Homeostasis and Induces Genotype‐Dependent Necrosis in *Theobroma cacao*

**DOI:** 10.1111/mpp.70316

**Published:** 2026-07-12

**Authors:** Taís Araújo Santos, Maria Luíza do Carmo Santos, Monaliza Macêdo Ferreira, Keilane Silva Farias, Carlos Henrique de Carvalho Neto, Ronan Xavier Corrêa, Uilson Vanderlei Lopes, Carlos Priminho Pirovani

**Affiliations:** ^1^ Departamento de Ciências Biológicas, Centro de Biotecnologia e Genética (CBG) Universidade Estadual de Santa Cruz (UESC) Ilhéus Brazil; ^2^ Embrapa Mandioca e Fruticultura (CNPMF) Cruz das Almas Brazil; ^3^ Centro de Pesquisas do Cacau (CEPEC) Comissão Executiva do Plano da Lavoura Cacaueira (CEPLAC) Itabuna Brazil

**Keywords:** frosty pod rot, fungal effector, necrosis, oxidative stress, proteomics, susceptibility marker

## Abstract

Frosty pod rot (FPR) is an invasive disease responsible for severe yield losses of cacao trees. In this study, we characterised the *Mr*B1 protein (BASIDIN1 from *Moniliophthora roreri*), a homologue of the BASIDIN effector protein from *M. perniciosa.* Recombinant *Mr*B1 (r*Mr*B1) induced morphological changes typical of necrosis in *Nicotiana benthamiana* cell suspensions, including cell swelling, reduced GFP (green fluorescent protein) fluorescence, plasma membrane damage, and electrolyte leakage. Proteomic analysis of the cells indicated that r*Mr*B1 treatment modulated proteins associated with cellular homeostasis, including reduced abundance of proteins related to energy metabolism and protein folding, such as BiP (Binding Immunoglobulin Protein), whose non‐detection was validated by western blotting. In cacao leaves, spraying with r*Mr*B1 (12.5 μM) resulted in necrotic lesions affecting 54% of the leaf area after 10 days, increasing to 77% in the presence of 0.5% urea. At a lower concentration (5 μM), the recombinant protein promoted hydrogen peroxide accumulation, supporting the induction of oxidative stress. The phenotypic response to r*Mr*B1 varied among cacao genotypes with different levels of resistance to FPR and witches' broom disease, with the susceptible Catongo genotype being the most affected, while resistant genotypes, such as ICS‐95 and Scavina‐6, did not show visible symptoms. These results position *Mr*B1 among the few functionally characterised effector proteins in the *M. roreri–Theobroma cacao* pathosystem and suggest that this effector may be further evaluated as a candidate marker of susceptibility in cacao breeding programmes.

## Graphical abstract

The effector *Mr*B1 induces cytotoxicity in plant cells through oxidative stress, membrane damage, protein folding collapse, and energy failure. These alterations compromise cellular integrity and result in necrosis.

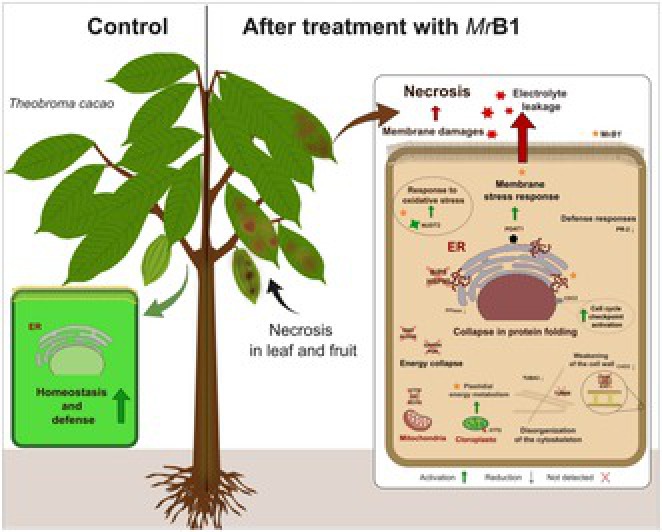

## 1 Introduction

Frosty pod rot (FPR), caused by the basidiomycete fungus *Moniliophthora roreri*, is a highly invasive disease that can lead to low yields and even total crop loss (Phillips‐Mora et al. 2015; Bailey et al. 2018). Due to its high potential for dissemination and aggressiveness, *M. roreri* is considered one of the main limiting factors of cacao production in affected regions (Phillips‐Mora et al. 2015; Johnson et al. 2017; Jiménez et al. 2021). Recently, the detection of new foci of the disease in Brazil (where the pathogen was previously classified as absent) has intensified concerns about its spread and impact on cacao farming (MAPA 2021, 2024).

The fungus has a hemibiotrophic life cycle, with two well‐defined stages. In the biotrophic phase, the fruits develop malformations. Subsequently, the transition to the necrotrophic phase occurs, characterised by rotting, sporulation, and tissue death within a few days (Evans 2007; Bailey et al. 2014).

Genomic and transcriptomic analyses of *M. roreri* have provided important insights into the molecular mechanisms underlying the biotrophic and necrotrophic phases, indicating that different sets of genes and proteins are phase‐specifically regulated (Meinhardt et al. 2014). During the biotrophic phase, upregulated proteins are associated with extracellular matrix degradation and fungal mycelium modulation, likely helping to mask the pathogen from plant defences. In the necrotrophic phase, secreted proteins protect the mycelium from plant defences while releasing enzymes and toxins that attack the cell wall, causing necrosis. In addition, proteomic analysis comparing spores and mycelium of *M. roreri* identified proteins involved in both developmental stages, including proteins related to metabolic processes, oxidation–reduction processes, biosynthesis, and protein folding (Zugaib et al. 2023).

To establish infection, the pathogen needs to employ different molecules at distinct stages of the disease, including effectors. The mode of action of effector proteins depends on the nature of the interaction between the pathogen and the host plant, and may favour infection or trigger defence responses (Santos et al. 2025). Studies have significantly advanced understanding of the functions of fungal effectors, demonstrating how they modulate the plant's immune response in different ways (Han et al. 2021; Gao et al. 2025; Li et al. 2026). Some effectors, such as the ethylene necrosis‐inducing protein of *Moniliophthora perniciosa* (*Mp*NEP2), contribute to the pathogenicity of the fungus by inducing cell death (Garcia et al. 2007). Others can suppress the plant's immune responses, aggravating the pathogen's virulence (Ma et al. 2021; Ebert et al. 2021).

The phylum Basidiomycota includes several species of phytopathogenic fungi whose effectors play a central role in the establishment of diseases. Although advances have been made in elucidating the action mechanisms of fungal effectors, the functional characterisation of these proteins in basidiomycetes remains less explored compared to ascomycetes, resulting in relevant gaps in current knowledge (Santos et al. 2025). BASIDIN from *M. perniciosa* exemplifies the potential of basidiomycete effectors in plant–pathogen interaction (Farias et al. 2023). Functional characterisation demonstrated that BASIDIN interferes with the host plant's defence system, inducing wilting and necrotic symptoms. Furthermore, the protein induces the production of hydrogen peroxide in leaf tissues, damaging the cell membrane and compromising the photosynthetic rate of tomato ( *Solanum lycopersicum* ).

Effectors likely play distinct roles in different stages of FPR disease. However, despite the importance of *M. roreri* as a cacao pathogen, research on the effector activity of this fungus is still incipient, although several candidate effector proteins have been identified in its genome and transcriptome (Barbosa et al. 2018; Nascimento et al. 2025). Effector proteins are fundamental in pathogen–host interactions, and the scarcity of studies on these molecules and their mechanisms of action denotes a significant gap in understanding the pathogenicity of the fungus.

In this context, we characterised a novel candidate effector protein, named *Mr*B1 (BASIDIN1 from *Moniliophthora roreri*), which exhibits sequence similarity to the BASIDIN of *M. perniciosa* . This study presents the characterisation of an effector that may be conserved among fungi of the genus *Moniliophthora*, providing new insights into the common pathogenicity strategies employed by members of this genus. We investigated the mechanism of action of *Mr*B1 in *Nicotiana benthamiana* cells and cacao ( *Theobroma cacao* ) tissues, the host plant of the fungus causing FPR.

## 2 Results

### 2.1 Characterisation of the Sequence, Expression and Structure of the *Mr*B1 Protein

**FIGURE 1:**
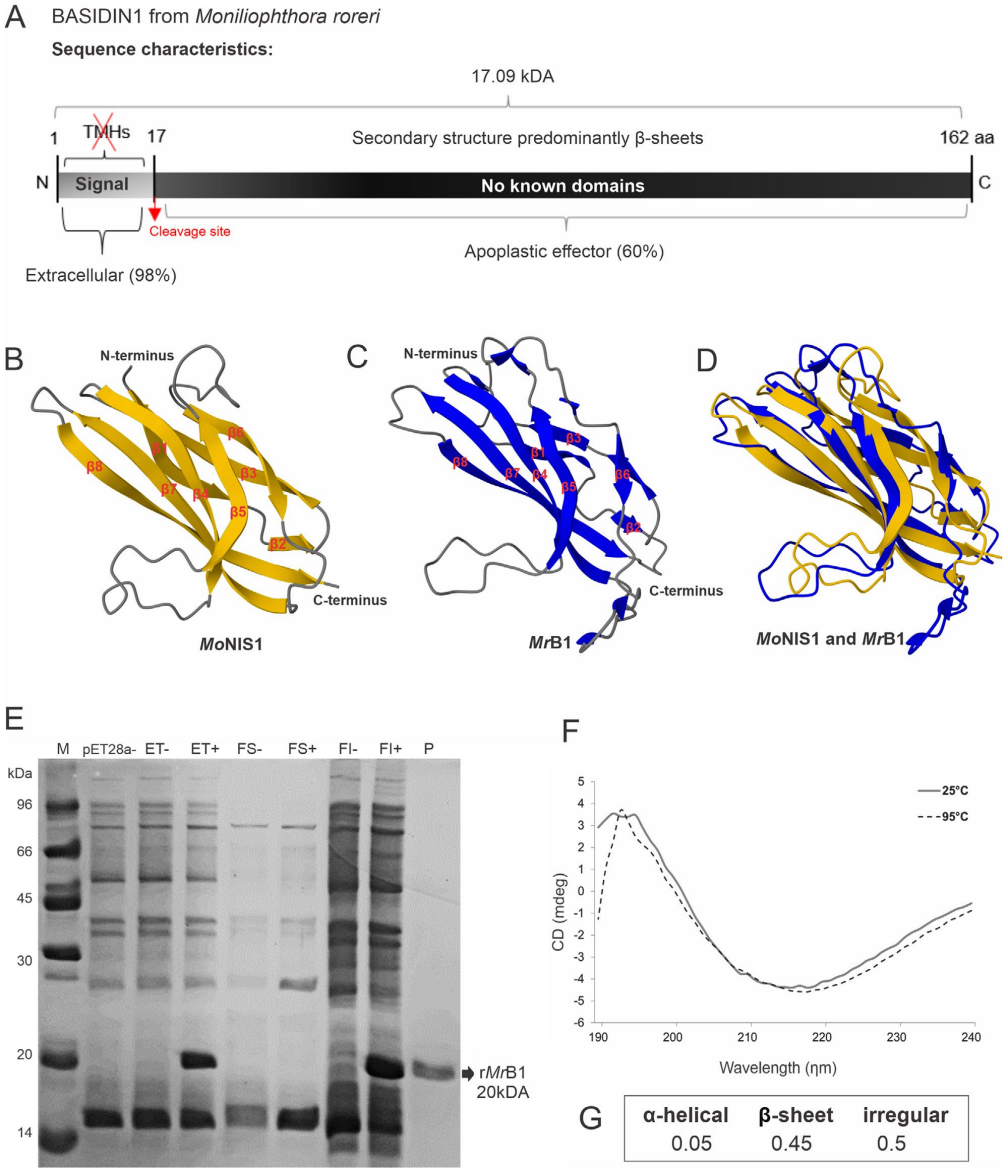
Characteristics of the sequence, structural analysis, expression, purification and circular dichroism (CD) spectra of r*Mr*B1. (A) In silico analyses. Signal: signal peptide, aa: amino acid, TMHs: transmembrane helices. (B) Three‐dimensional structure of *Mo*NIS1 (PDB ID: 8XGL‐B), a necrosis‐inducing secreted protein 1 (NIS1) family effector from *Magnaporthe oryzae*, obtained by X‐ray diffraction, showing an eight‐stranded β‐barrel arrangement. (C) Predicted three‐dimensional model of *Mr*B1, with the same eight‐stranded β‐barrel arrangement. (D) Structural superposition of *Mo*NIS1 and *Mr*B1 (*Z*‐score of 8.4 and root mean square deviation [RMSD] of 3.2 Å). (E) r*Mr*B1 expression profile analysed by SDS‐PAGE, highlighting the purified 20 kDa band corresponding to the recombinant protein. (M): Marker; pET28a−: Total extract of cells transformed with pET28a without insert; (ET−): Total extract without IPTG (negative control); (ET+): Total extract with IPTG (pET28a + insert); (FS−): Soluble fraction without IPTG; (FS+): Soluble fraction with IPTG; (FI−): Insoluble fraction without IPTG; (FI+): Insoluble fraction with IPTG; (P): purified protein. (F) CD spectra of the protein at 25°C and 95°C, demonstrating thermostability. (G) Analysis of the secondary structure of r*Mr*B1 based on CD spectra.

The *Mr*B1 protein has a predicted molecular weight of 17.09 kDa, is composed of 162 amino acids, and contains a predicted N‐terminal signal peptide with a cleavage site at amino acid 17 (Figure 1A). No transmembrane helices were identified, and subcellular localisation prediction indicated a high probability of extracellular localisation (98%). Sequence‐based analysis did not identify any known conserved domains in *Mr*B1 but indicated two disordered regions (Figure S1). Effector prediction classified *Mr*B1 as a putative apoplastic effector.

Structural analysis of the predicted *Mr*B1 model using the DALI server identified *Mo*NIS1 from *Magnaporthe oryzae* (PDB ID: 8XGL‐B), a necrosis‐inducing secreted protein 1 (NIS1) family effector, as a structural homologue of *Mr*B1 (Figure 1B,C). The alignment showed a *Z*‐score of 8.4, above the statistical significance threshold of the server (*Z* > 2), with a root mean square deviation (RMSD) of 3.2 Å over 104 structurally equivalent residues, despite low sequence identity (13%). Structural superposition showed that both proteins share an eight‐stranded β‐barrel arrangement, with conservation mainly concentrated in the central structural core (Figure 1D). The expression of recombinant *Mr*B1 (r*Mr*B1), with the addition of six histidine residues from the vector, resulted in a protein of approximately 20 kDa after induction with IPTG (Figure 1E). Protein identity was confirmed by mass spectrometry after trypsin digestion of the 20 kDa band from the gel (Figure S2).

Although attempts to purify r*Mr*B1 from the soluble fraction failed due to low accumulation in this fraction, the protein was obtained in higher yields by isolating inclusion bodies (insoluble fraction) from *Escherichia coli* cells and solubilizing them with urea. The recombinant protein was then purified by affinity chromatography from the insoluble fraction of the bacterial extract. SDS‐PAGE analysis revealed a single 20 kDa band corresponding to the purified protein (Figure 1E).

The secondary structure of r*Mr*B1 was analysed by circular dichroism (CD) spectroscopy (Figure 1F). The spectrum showed a positive peak at 194 nm and a negative peak at 218 nm. The spectra obtained at 95°C were similar to those obtained at 25°C, indicating that the protein maintained its conformation after heating. Quantitative analysis of the α‐helix and β‐sheet content, from the CD spectra, demonstrated that the protein is composed of 45% β‐sheets, 5% α‐helices, and 50% irregular structures (Figure 1G), consistent with the β‐sheet predominance observed in the predicted structural model.

### 2.2 Analysis of the Effect of r*Mr*B1 on *N. benthamiana* Cells Expressing GFP

**FIGURE 2:**
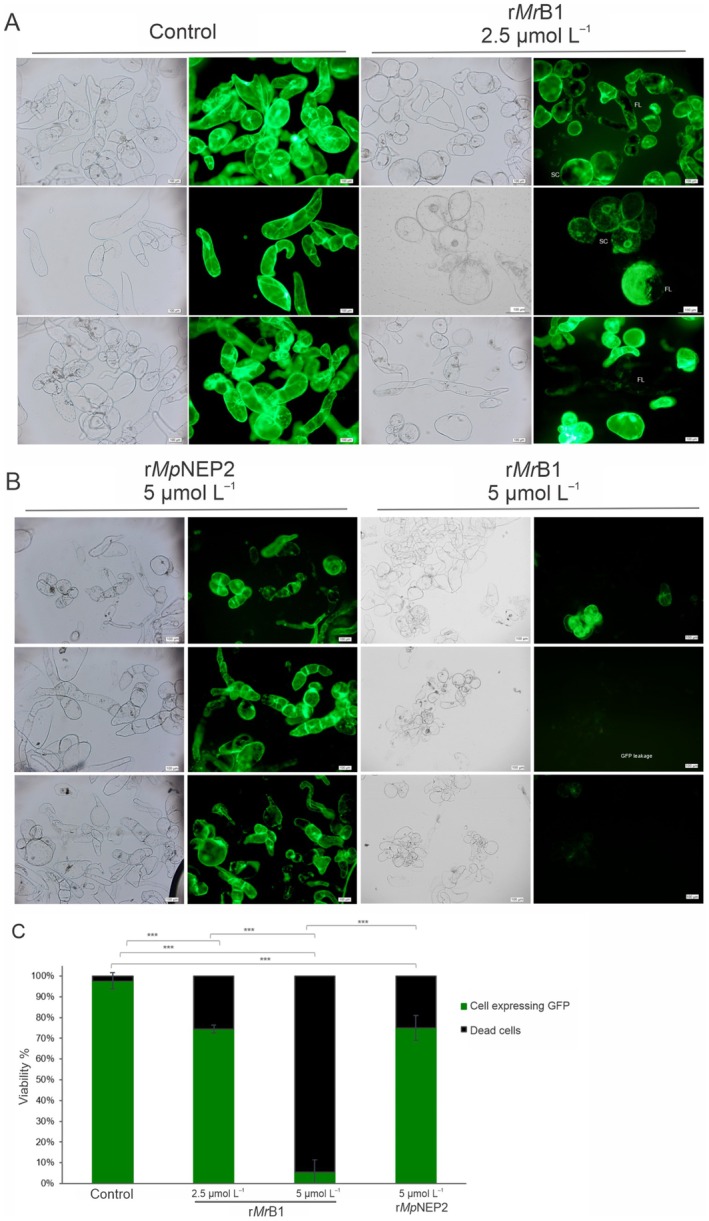
Bright‐field microscopy and epifluorescence images and quantitative analysis of *Nicotiana benthamiana* cell viability under different treatment conditions after 1 h. GFP fluorescence was used as a marker of cell viability. (A) Cells treated with 10 mM Tris–HCl buffer (control), showing mostly intact cells, and cells treated with 2.5 μM r*Mr*B1, showing evident morphological changes. (SC): Swollen cells; (FL): Loss of fluorescence. (B) Cells treated with 5 μM r*Mp*NEP2 and r*Mr*B1, demonstrating loss of fluorescence. (C) Percentage of cells expressing GFP and dead cells after the different treatments. Statistically significant differences between pairs are indicated in the graphs: ***p < 0.01. Scale bars correspond to 100 μm.

Cell viability was evaluated by quantifying the proportion of *N. benthamiana* cells expressing GFP (live cells) and cells not expressing GFP (dead cells) under different treatment conditions after 1 h (Figure 2, Table S1). In the control, a high percentage of cells expressing GFP (98%) was observed, with only 2% dead cells (Figure 2A,C). With treatment of 2.5 μM r*Mr*B1, there was a significant reduction of GFP expression, with 26% of the cells dead. The cells treated with the protein showed evident morphological changes, becoming swollen and irregular, with reduced fluorescence (Figure S3). The control cells maintained a more regular and structured shape. The effect of r*Mr*B1 at 5 μM was even more pronounced, with 95% of the cells dead, evidenced by the loss of GFP inside the cells (Figure 2B,C). In comparison, when cells were treated with 5 μM of r*Mp*NEP2, only 25% were dead.

### 2.3 rMrB1 Induces Cell Membrane Damage in *N. benthamiana*

**FIGURE 3:**
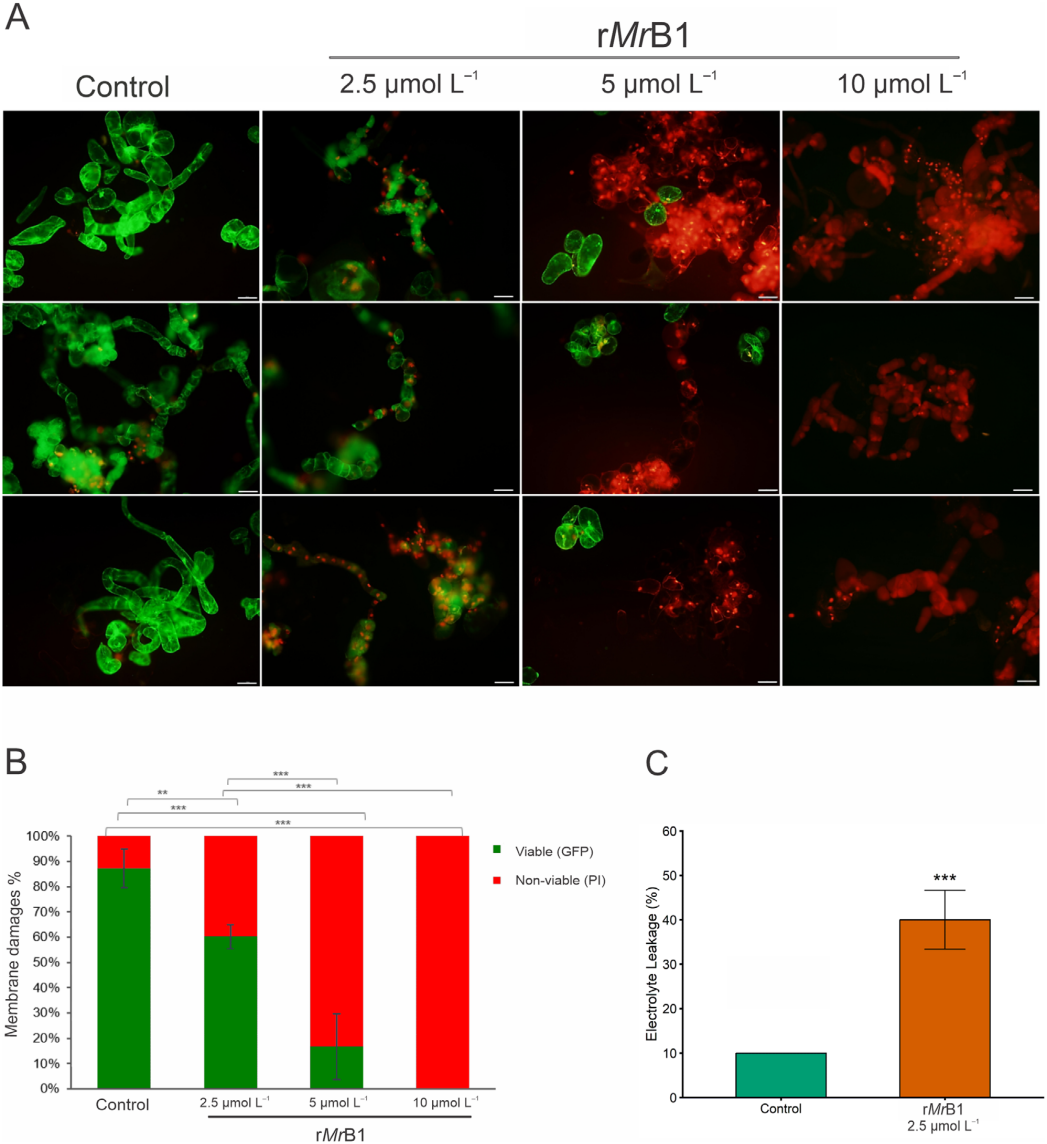
Epifluorescence microscopy images and quantitative analysis of cell membrane damage in *Nicotiana benthamiana*. (A) Cells were treated with 10 mM Tris–HCl buffer and different concentrations of r*Mr*B1 for 1 h, followed by staining with 20 μg mL^−1^ propidium iodide (PI). PI staining indicates plasma membrane permeabilisation. Viable cells do not exhibit red fluorescence (negative for PI), while nonviable cells exhibit red fluorescence (positive for PI). (B) Percentage of viable and nonviable cells, determined based on fluorescence. (C) Electrolyte leakage assay in cells in suspension 24 h after treatment with r*Mr*B1. Statistically significant differences are indicated in the graphs: **p < 0.05, ***p < 0.01.

The results revealed that the protein caused significant damage to the cell membrane (Figure 3A,B). This effect was evidenced by the use of propidium iodide (PI) in *N. benthamiana* cell cultures expressing GFP, where the presence of red fluorescence indicates membrane damage. In the control group, 87% of the cells remained viable, while 13% became nonviable. When cells were treated with r*Mr*B1 at a concentration of 2.5 μM, cell viability decreased, with 40% of the cells becoming nonviable. When the concentration was increased to 5 μM, 83% of the cells became nonviable. At the highest concentration tested, 10 μM, all cells became nonviable.

Furthermore, treatment with the protein at a concentration of 2.5 μM for 24 h resulted in a significant increase in the electrical conductivity of *N. benthamiana* cells in suspension compared to the control (Figure 3C). The mean percentage of electrolyte leakage was 9.8% in the control and 40% in the r*Mr*B1 treatment (*p* < 0.05).

### 2.4 rMrB1 Modulates the Proteomic Profile of *N. benthamiana*

**FIGURE 4:**
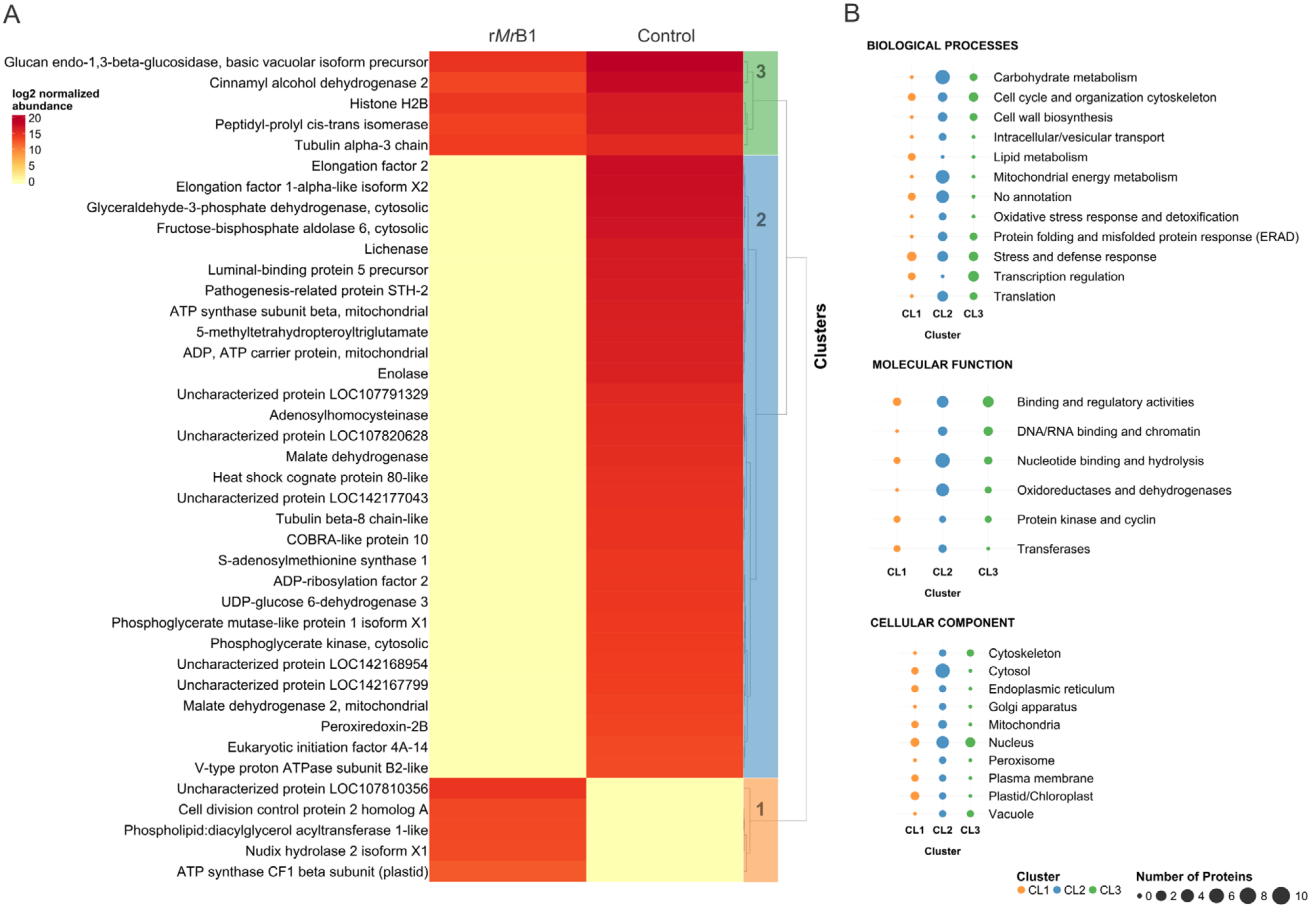
Functional profile of proteins identified in *Nicotiana benthamiana* cells under control and r*Mr*B1‐treated conditions. Proteins were identified by mass spectrometry after treatment with r*Mr*B1 or buffer control. After applying quality criteria (false discovery rate [FDR] < 1%, score > 5, and peak‐scored intensity [SPI] > 60%), 40 proteins showed treatment‐associated differences. (A) Heatmap of protein intensities transformed into log_2_(*x* + 1) and organised by hierarchical clustering using Euclidean distance and the complete linkage method. Cluster 1 includes proteins detected exclusively after r*Mr*B1 treatment; cluster 2 includes proteins detected exclusively in the control; and cluster 3 includes proteins detected in both conditions, with reduced abundance after r*Mr*B1 treatment. The colour scale represents relative protein abundance. (B) Functional annotation of the proteins in each cluster based on Gene Ontology terms (Biological Process, Molecular Function, and Cellular Component). The size of the bubbles indicates the number of proteins per functional category, and the colours correspond to the clusters defined in the heatmap.

Protein extraction efficiency was confirmed by SDS‐PAGE (Figure S4). Mass spectrometry analysis identified 245 proteins in *N. benthamiana* cells under control conditions and 216 proteins in r*Mr*B1‐treated cells. After applying quality criteria (false positive rate [FDR] < 1%, score > 5, and peak‐scored intensity [SPI] > 60%), a set of 40 proteins showed alterations associated with the treatment (Table S2). Among these, 30 proteins were exclusively detected under control conditions, five proteins were exclusively detected after treatment with r*Mr*B1, and five proteins were detected in both conditions (showing reduced abundance after the r*Mr*B1 treatment compared to the control) (Figure 4A).

Treatment with r*Mr*B1 revealed the following unique proteins: cell division control protein 2 homologue A; ATP synthase CF1 β subunit (plastidial); phospholipid: diacylglycerol acyltransferase 1‐like; nudix hydrolase 2 isoform X1; and the uncharacterised protein LOC107810356. Control‐unique proteins included chaperones such as heat shock cognate protein 80‐like and luminal‐binding protein 5 (BiP5), as well as enzymes associated with energy metabolism, such as malate dehydrogenase, ATP synthase subunit beta, glyceraldehyde‐3‐phosphate dehydrogenase, and proteins related to translation, cytoskeleton, and metabolic maintenance. Five common proteins were less abundant after treatment with r*Mr*B1, namely tubulin α‐3 chain, histone H2B, peptidyl‐prolyl cis‐trans isomerase, cinnamyl alcohol dehydrogenase 2, and glucan endo‐1,3‐β‐glucosidase.

Hierarchical analysis revealed three main clusters (Figure 4A,B). Cluster 1, formed by proteins unique to the r*Mr*B1 treatment, included proteins related to stress and defence response, lipid metabolism, and regulatory functions. Cluster 2, composed of proteins unique to the control group, was enriched in biological processes associated with mitochondrial energy metabolism, carbohydrate metabolism, translation, protein folding, stress response, cellular structural organisation, and maintenance of cellular homeostasis. Cluster 3 grouped common proteins with reduced abundance after the r*Mr*B1 treatment, associated with transcriptional regulation, cell cycling, cytoskeleton organisation, translation, and stress response.

Regarding molecular function, cluster 1 concentrated proteins with transfer activities and regulatory functions. Cluster 2 was enriched by proteins involved in nucleotide binding and hydrolysis and in redox activities, while cluster 3 was characterised by proteins associated with DNA/RNA binding and transcriptional regulation. In relation to the cellular component, cluster 2 showed a predominance of cytosolic, nuclear and mitochondrial proteins, and clusters 1 and 3 mainly contained nuclear proteins, with associations to plastids, vacuoles and the cytoskeleton.

### 2.5 The Protein–Protein Interaction Network Reveals Functional Clusters Differentially Affected by r*Mr*B1

**FIGURE 5:**
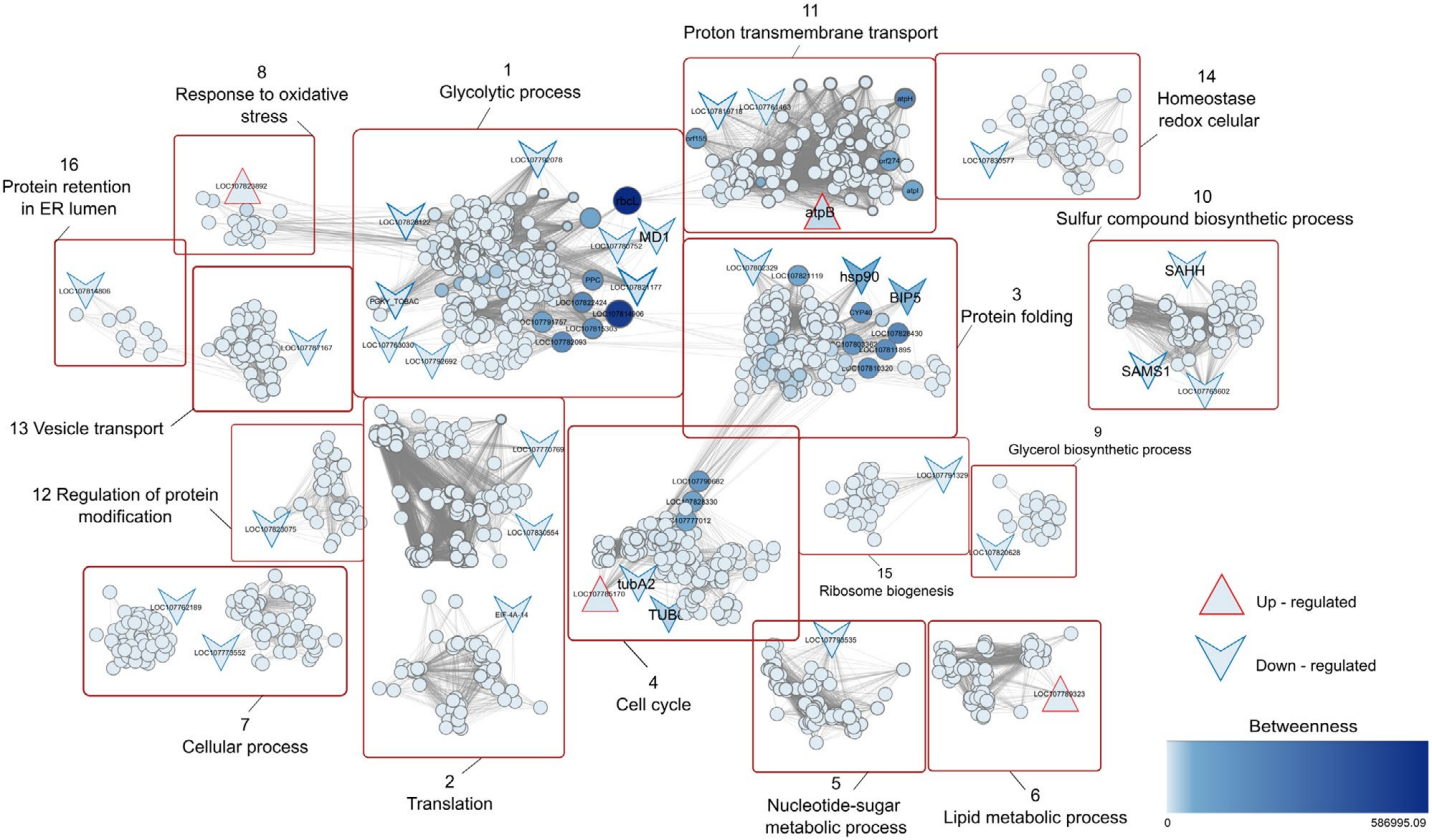
Protein–protein interaction (PPI) network of proteins detected in *Nicotiana benthamiana* cells under control and r*Mr*B1‐treated conditions. The network was constructed using proteins identified as exclusive to the control, exclusive to the r*Mr*B1 treatment, or reduced after r*Mr*B1 treatment, based on the proteomic analysis. The PPI network has 1944 nodes and 39,601 edges, organised into 16 functional clusters. Triangles with blue borders represent proteins exclusive to the control or downregulated in the r*Mr*B1 treatment, while triangles with red borders indicate proteins exclusive to the r*Mr*B1 treatment. The main functional clusters were associated with glycolytic process, translation, protein folding, cell cycle, oxidative stress response, lipid metabolism, and transmembrane proton transport. Node colour intensity reflects the betweenness values, highlighting proteins classified as bottlenecks, while the thickness of the node edges indicates the degree of connectivity, allowing the identification of hub proteins within the network.

The protein–protein interaction (PPI) network, constructed from 34 proteins that met the minimum confidence criteria, displayed a highly connected architecture, comprising 1944 nodes and 39,601 interactions organised into 16 functionally enriched clusters, all showing more interactions than expected by chance (PPI enrichment *p*‐value < 1e−16) (Figure 5). The clusters with the highest number of proteins and the highest density of interactions were cluster 1 with 407 nodes associated with the glycolytic process, cluster 2 with 229 nodes related to translation, and cluster 3 with 241 nodes associated with protein folding, all predominantly formed by proteins downregulated in the r*Mr*B1 treatment. Proteins upregulated in the r*Mr*B1 treatment were present in four clusters: cluster 4, with 202 nodes associated with the cell cycle; cluster 8, with 21 nodes related to the response to oxidative stress; cluster 6, with 84 nodes involved in lipid metabolism; and cluster 11, with 185 nodes related to transmembrane proton transport. Topological analysis identified 1336 proteins classified as hubs, predominantly associated with energy metabolism, and 444 proteins classified as bottlenecks, characterised by high betweenness values and acting as central mediators of information flows between network clusters. Among the bottlenecks, proteins related to energy and carbon metabolism (rbcL, LOC107814906, LOC107779254, atpH, atpI, atpF and PPC), chaperone‐dependent regulation (HSP90 and CYP40), and protein processing and folding in the endoplasmic reticulum (ER) (BiP5) stood out. These results provide an integrated description of the network's structural organisation, the distribution of up‐ and downregulated proteins, and the key central elements responsible for connectivity and functional integration among the clusters affected by r*Mr*B1.

### 2.6 Effect of r*Mr*B1 on BiP Accumulation in *N. benthamiana*

**FIGURE 6:**
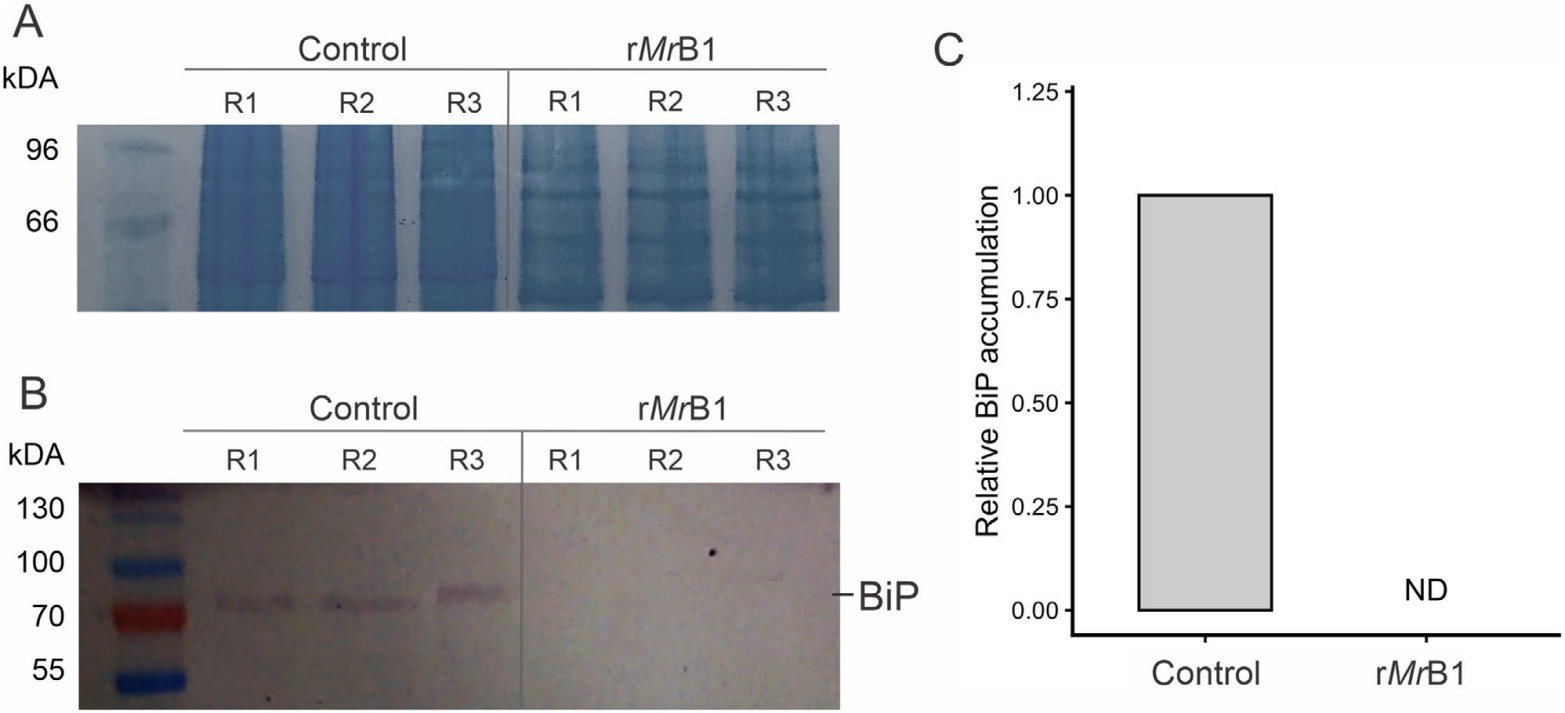
Validation of BiP non‐detection in *Nicotiana benthamiana* cells treated with r*Mr*B1. (A) Mirror gel stained with Coomassie blue showing the total protein profile of control and r*Mr*B1‐treated samples, in triplicate, confirming equivalent loading. (B) Immunodetection of the BiP protein by western blotting, showing BiP detection in the control samples and no detectable signal in the rMrB1‐treated samples. (C) Relative quantification of the BiP signal, expressed relative to the control (1.0), indicating a signal below the detection limit in the r*Mr*B1‐treated samples (ND).

To validate the data obtained by proteomic analysis, which indicated impairment of the ER chaperone BiP (Binding Immunoglobulin Protein) after treatment with r*Mr*B1, we performed immunodetection of BiP in total protein extracts of cells treated with r*Mr*B1 and the control group. A Coomassie blue‐stained parallel gel confirmed the integrity of the samples and the equivalence of protein loading between treatments (Figure 6A). Western blot analysis detected BiP in the control samples, whereas no detectable signal was observed in r*Mr*B1‐treated samples, indicating non‐detection of this protein after treatment and supporting the proteomic results (Figure 6B,C).

### 2.7 Functional Analyses of r*Mr*B1 in *T. cacao*

**FIGURE 7:**
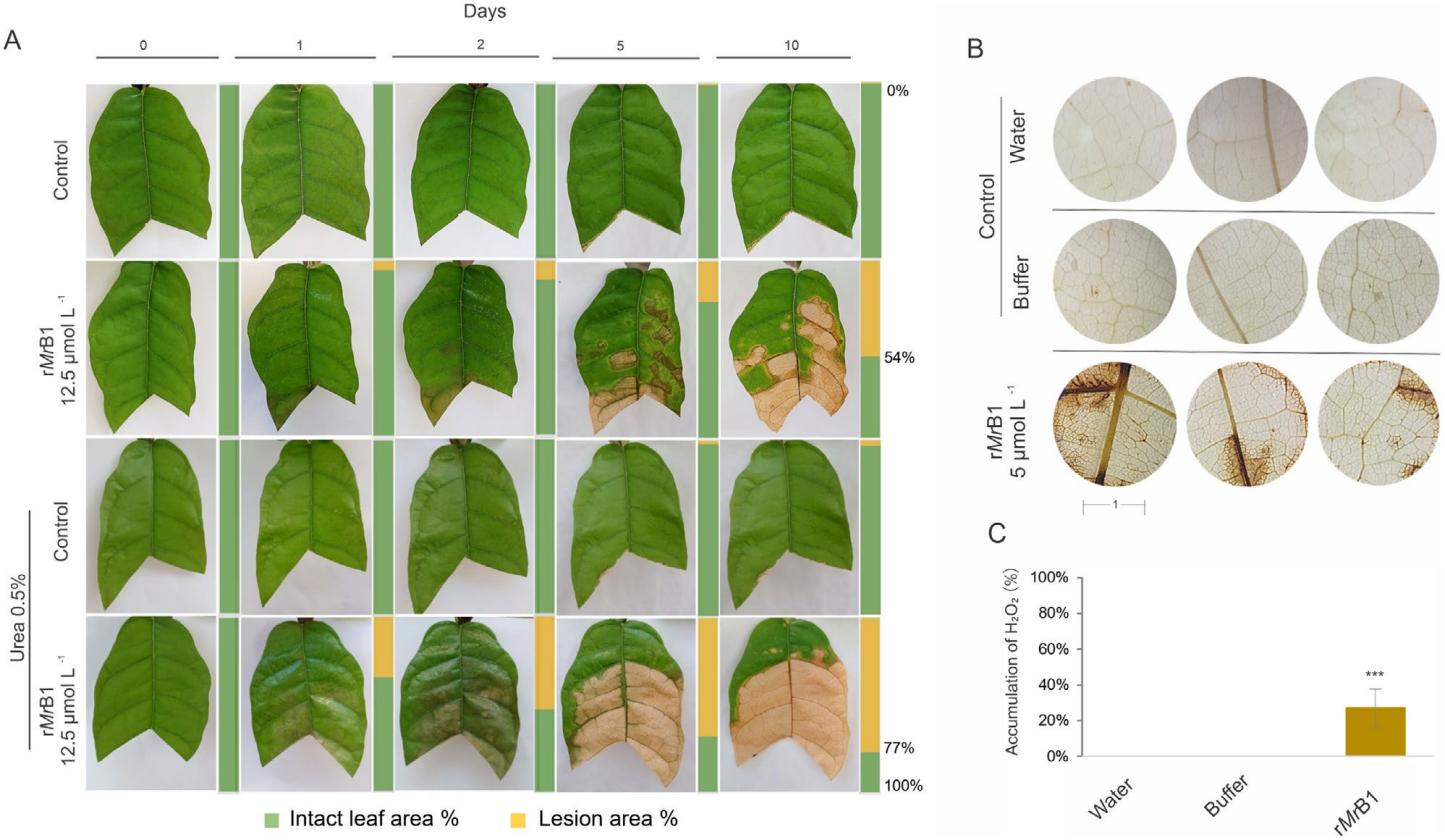
Effect of r*Mr*B1 on cacao leaves (Catongo genotype). (A) Leaves treated with 10 mM Tris–HCl buffer (with and without urea) showed no visible symptoms. On the other hand, leaves treated with r*Mr*B1 (with and without urea) at a concentration of 12.5 μM showed necrotic lesions after 10 days. The bar graph on the right side of the leaf shows the percentage of the lesioned area in relation to the total leaf area, quantified in pixels using ImageJ2. (B) Detection of hydrogen peroxide (H_2_O_2_) in leaf discs by staining with 3,3′‐diaminobenzidine (DAB). Leaf discs treated with r*Mr*B1 showed increased H_2_O_2_ accumulation compared to controls. (C) Quantification of leaf area with H_2_O_2_ accumulation in relation to the total area of the leaf disc, quantified in pixels using ImageJ2.

Cacao leaves (Catongo genotype) treated with r*Mr*B1 showed visible necrotic lesions over time, with the lesions becoming more evident on the 10th day (Figure 7A). In contrast, leaves treated with buffer (control) maintained their integrity and coloration during the observation period. The bar graph to the right of each leaf shows a progressive increase in the percentage of lesions in the leaves treated with the protein at 12.5 μM, reaching 54% on the tenth day. The results also demonstrated that after treatment with r*Mr*B1 + urea, 77% of the leaf area was compromised by necrotic lesions. In contrast, treatment with buffer + urea (control) resulted in a minimal percentage of lesions (less than 1%). These results indicate that the differences observed in cacao leaves were associated with the application of the protein rather than with the buffer or urea treatment. Furthermore, the injection of r*Mr*B1 (5 μM) into cacao fruits resulted in the formation of necrotic spots 5 days after inoculation, corroborating the effects observed in leaves and plant cells (Figure S5).

Oxidative stress in cacao leaves was evaluated by detecting H_2_O_2_ using staining with 3,3′‐diaminobenzidine (DAB). Leaf discs treated with 5 μM of the protein showed brown colouration, resulting from the polymerisation of DAB, indicating an increase in H_2_O_2_ accumulation (Figure 7B). Quantification revealed that discs treated with r*Mr*B1 induced a greater accumulation of H_2_O_2_ compared to the negative controls (water and buffer) (Figure 7C and Table S3).

### 2.8 Differential Responses Induced by r*Mr*B1 in Cacao Genotypes

**FIGURE 8:**
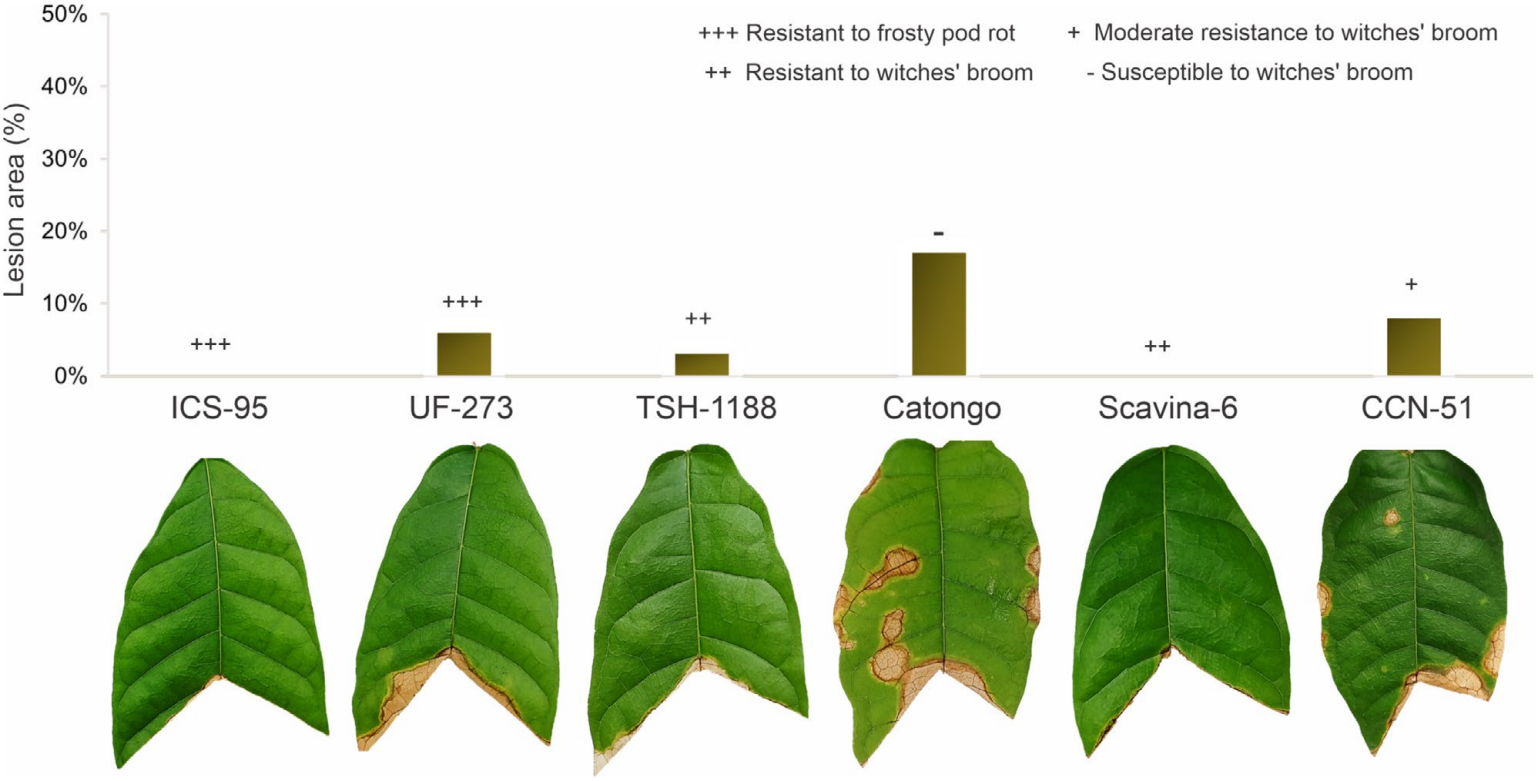
Effect of r*Mr*B1 (5 μM) on six cacao genotypes 10 days after treatment. Genotypes with resistance to frosty pod rot (ICS‐95 and UF‐273) and genotypes with different levels of resistance to witches' broom disease (WBD) were evaluated: TSH‐1188 and Scavina‐6 (resistance patterns), CCN‐51 (moderate resistance), and Catongo (susceptible). The bar graph denotes the percentage of damaged area in relation to the total leaf area, highlighting the responses of the different genotypes treated with r*Mr*B1.

The effect of r*Mr*B1 on leaves of cacao genotypes with varying levels of resistance to FPR and witches' broom disease (WBD) was evaluated using a reduced concentration of the protein (5 μM). The experiment was conducted in two independent replicates, showing consistent results. The data presented corresponded to the second experimental replicate. After treatment, a differential response was observed among the genotypes, according to their resistance to these diseases (Figure 8). Genotypes ICS‐95 and Scavina‐6 did not show visible lesions. In contrast, genotypes TSH‐1188 and UF‐273 exhibited chlorotic spots on the edges of the leaf discs, corresponding to 3% and 6% of the affected area, respectively. Genotypes CCN‐51 and Catongo showed the most pronounced damage, with 8% and 17% necrotic area, respectively.

## 3 Discussion

The mechanism by which *M. roreri* penetrates cacao fruits is not yet fully understood, but effectors are key factors in pathogenicity. Recently, functional analysis involving potential *M. roreri* effectors showed that these proteins can induce wilting, drying and necrosis in leaf tissues, reinforcing the relevance of effectors in this pathosystem (Nascimento et al. 2025). In this context, we characterised a new *M. roreri* protein, with no previously known function, but with 92% sequence similarity to BASIDIN from *M. perniciosa* (Farias et al. 2023). The choice to study the homologous effector protein, *Mr*B1, was based on the premise that the conservation of sequences between proteins from different pathogens might indicate the preservation of biological functions essential for the establishment of diseases.

### 3.1 r*Mr*B1 Reduces Cellular Integrity by Causing Damage to the Membranes of *N. benthamiana*

To evaluate the mechanism of action of r*Mr*B1, we employed the *N. benthamiana* cell suspension assay to determine GFP expression. The assay indicated that the protein impaired cell integrity within 1 h of treatment. Cells treated with r*Mr*B1 exhibited swelling and morphological alterations, with a more irregular appearance compared to the control, in addition to the release of GFP into the extracellular medium. These alterations are typical characteristics of necrosis, a process in which cells can undergo increased volume, rupture of the plasma membrane, and loss of intracellular content (Van Doorn et al. 2011).

Studies have demonstrated that treatment with *Mp*NEP2 rapidly reduces the number of *N. benthamiana* cells expressing GFP, in addition to inducing cytological changes associated with the necrosis process (Villela‐Dias et al. 2014; Do Carmo Santos et al. 2024). In our study, r*Mr*B1 induced a significantly higher cell death rate (94%) compared to r*Mp*NEP2 (25%). Although both induced necrosis, each protein may have a unique set of interactions with cellular components, which can influence the extent and speed of induced necrosis.

The plasma membrane plays a crucial role in separating the intracellular from the extracellular environment by regulating the transport of substances and detecting a variety of signals. Therefore, damage to the membrane can compromise cellular integrity, resulting in cell death or dysfunction of essential physiological processes (Horn and Jaiswal 2019). To assess membrane damage, we used PI, a dye widely employed to evaluate cell viability (Rosenberg et al. 2019). PI penetrates cells with damaged membranes and binds to DNA, emitting fluorescence.

Analysis of the effects of r*Mr*B1 revealed its cytotoxic potential, with evidence of plasma membrane damage consistent with necrotrophic effectors. Cell viability decreased with increasing r*Mr*B1 concentration, as evidenced by increased PI fluorescence. At the maximum concentration tested (10 μM), all cells became nonviable. Furthermore, the significant increase in electrolyte leakage in cells treated with the protein confirmed that it causes significant damage to the plasma membrane, leading to loss of intracellular content and cell death. Nep1‐like proteins (NLPs), secreted by phytopathogens, were initially considered the only protein effectors that cause plasma membrane damage (Pirc et al. 2023). However, it has been shown that other effectors can also affect plasma membrane integrity in plant cells (Zhu et al. 2022).

More broadly, studies involving fungal models have demonstrated that loss of membrane integrity can integrate into regulated cell death pathways, revealing interdependence between physical damage and biochemical signals during the progression of cell death (Wengler and Talbot 2025). Therefore, proteomic studies of interactions involving effectors are crucial to reveal the biochemical and structural pathways modulated in response to the effector (Day et al. 2015).

### 3.2 r*Mr*B1 Modulates Proteins Associated With Cellular Homeostasis in *N. benthamiana*

In this study, the integration of cytological and proteomic data indicated that structural alterations were accompanied by changes in proteins associated with cellular homeostasis. The combined analysis of proteins unique to both conditions, of proteins reduced after treatment with r*Mr*B1, and of the PPI network indicated that r*Mr*B1 primarily affects membrane integrity, cell cycle, stress responses, energy metabolism, and protein folding.

The proteins detected exclusively after r*Mr*B1 treatment were associated with cellular responses related to metabolic, oxidative, and membrane stress in *N. benthamiana*. Elicitors and effectors can induce alterations in plant lipid metabolism, including the accumulation of triacylglycerols (Schieferle et al. 2021). Phospholipid: diacylglycerol acyltransferase 1‐like (PDAT1‐like), an enzyme involved in membrane stress conditions, promotes lipid redirection and bilayer reorganisation in the ER to mitigate disturbances (Zhao et al. 2025). The detection of PDAT1 after treatment suggests lipid bilayer remodelling in response to membrane damage associated with r*Mr*B1, corroborating the morphological and biochemical evidence of compromised membrane integrity.

Nudix Hydrolase 2 was detected only after r*Mr*B1 treatment and was associated with an oxidative stress cluster in the PPI network, suggesting modulation of antioxidant responses. Recent studies have shown that fungal effectors can manipulate reactive oxygen species (ROS) homeostasis to induce plant cell death. For example, PdCDIE1 (*Penicillium digitatum* Cell Death‐Inducing Effector 1) interacts with Hsp70, disrupting CaM binding and promoting ROS‐dependent cell death, indicating that cytotoxic effectors can modulate plant redox responses during necrosis (Lin et al. 2025). The presence of Cell Division Control Protein 2 homologue, a cyclin‐dependent kinase (CDK) involved in cell cycle progression, also suggests modulation of cell cycle‐related processes. In plants, cell cycle progression under stress is controlled by CDKs and cyclins, and the activation of checkpoints allows the cell to pause the cycle for repair or, if the damage is irreversible, leads to cycle arrest or cell death (Qi and Zhang 2020).

The plastidial ATP synthase β subunit (CF1), identified after treatment with the protein, may reflect adjustments in energy metabolism and redox balance during the cellular response (Yang et al. 2020). In contrast, several enzymes, including malate dehydrogenase, glyceraldehyde‐3‐phosphate dehydrogenase (GAPDH), enolase, phosphoglycerate kinase, and aldolase, were not detected in treated cells, indicating modulation of proteins associated with mitochondrial respiration and glycolysis. This modulation is consistent with previous reports of metabolic reprogramming during pathogen‐induced cell death in plants (Colombatti et al. 2014; Henry et al. 2015; Yang et al. 2022; Wang et al. 2022).

During interactions with fungal pathogens, the host cell cytoskeleton is remodelled and becomes a target for manipulation by molecules secreted by the pathogen, a process that impairs immune responses and favours the progression of infection (Sinha et al. 2024). In the present study, the non‐detection of tubulins, elongation factors and ADP‐ribosylation factor 2 (ARF2) in cells treated with the protein suggests cytoskeletal disorganisation and altered ARF2‐dependent vesicular trafficking. Proteins involved in cell wall biosynthesis, such as COBRA‐like and UDP‐glucose 6‐dehydrogenase 3, were also not recovered in treated cells, suggesting changes in extracellular matrix‐related processes. Furthermore, the decreased abundance of cinnamyl alcohol dehydrogenase 2 (CAD2) reinforces this vulnerability. When affected by fungal effectors, the cell wall becomes more susceptible to damage, weakening the plant's ability to withstand stress (Gu et al. 2024).

Effectors suppress immune responses and reprogramme host physiology to facilitate colonisation and promote pathogen virulence (Cai et al. 2023). Consistent with this pattern, pathogenesis‐related protein STH‐2 was detected only in the control, and glucan endo‐1,3‐β‐glucosidase showed decreased abundance after r*Mr*B1 treatment, indicating possible suppression of basal defence pathways. Furthermore, proteins involved in protein folding were not detected in treated cells, suggesting that cellular maintenance mechanisms may be affected. Analysis of the PPI network reinforces this interpretation, showing that clusters enriched with downregulated proteins are associated with glycolysis, translation, and protein folding. BiP5 and HSP90, detected only in the control, were identified as network bottlenecks, suggesting their relevance in the cellular response affected by r*Mr*B1.

BiP, belonging to the 70 kDa heat shock protein (HSP70) family, acts as a central chaperone in the ER lumen, facilitating the correct folding of newly synthesised proteins and preventing protein aggregation (Jing and Wang 2020). In this study, the non‐detection of BiP after r*Mr*B1 application was confirmed by western blotting, suggesting that ER‐associated protein folding may be affected. Plant pathogens use effectors to interfere with essential cellular processes, such as ER disorganisation, triggering stress, and in more intense cases, cell death (Liebrand et al. 2014; Liu et al. 2025).

As a master regulator of the unfolded protein response (UPR), BiP is essential for restoring ER homeostasis, and its non‐detection after treatment may increase cellular vulnerability and potentially contribute to cell death (Ducloy et al. 2024; Marzari et al. 2025). BiP‐related processes are relevant in plant–pathogen interactions. In *S. lycopersicum* , BiP overexpression increased resistance to *M. perniciosa* , with higher PR protein accumulation, enhanced antioxidant activity, and reduced ROS levels (Alcântara et al. 2025). In contrast, the phytopathogen *Ustilago maydis* depends on a functional UPR to maintain ER integrity and ensure efficient secretion of effector proteins during the biotrophic interaction, allowing the pathogen to effectively suppress host defenses and establish a compatible interaction (Pinter et al. 2019). Similarly, *Phytophthora sojae* secretes the effector PsAvh262, which prevents BiP degradation and helps maintain cell viability during the initial phase of infection (Jing et al. 2016). Silencing *PsAvh262* does not induce BiP expression, resulting in exacerbated cell death. In the case of r*Mr*B1, the non‐detection of BiP suggests that ER‐associated proteostasis may be affected during the cellular response induced by the protein, potentially contributing to cellular disorganisation and necrosis. This type of disorganisation is consistent with necrotrophic strategies or necrotrophic phases of hemibiotrophs, highlighting the importance of BiP manipulation in plant–pathogen interactions.

The reduced detection of peptidyl‐prolyl cis‐trans isomerase (PPIase) after r*Mr*B1 treatment further supports the involvement of proteostasis‐related processes in the cellular response induced by this effector. PPIases are known targets of pathogen effectors, such as PcAvr3a12, an RXLR effector of *Phytophthora capsici* from the Avr3a family, which suppresses ER‐dependent immunity by inhibiting the host plant's PPIase (Fan et al. 2018). Furthermore, when comparing our results with the proteomic study of *N. benthamiana* cells treated with NEP2 from *M. perniciosa* (Villela‐Dias et al. 2014), we observed a similar pattern of downregulation of proteins associated with protein folding, reinforcing the existence of common mechanisms for inducing necrosis in plant cells by *Moniliophthora* effectors.

### 3.3 r*Mr*B1 Induces Necrotic Lesions and Oxidative Stress in *T. cacao*

Although *N. benthamiana* is widely used for effector characterisation, its responses may not fully reflect those occurring in *T. cacao* . Therefore, we evaluated whether purified r*Mr*B1 also induces symptoms in cacao tissues. Ten days after treatment with the protein (12.5 μM), necrotic spots affected 54% of the leaf area, indicating that this *M. roreri* protein can induce symptoms in cacao leaves. Several cell‐death‐inducing proteins (CDIPs) have been characterised in fungal pathogens (Sabnam et al. 2023). Similar necrosis‐inducing activity has been described for *Mp*NEP1 and *Mp*NEP2 from *M. perniciosa* in plant cells and cacao leaves (Garcia et al. 2007; Villela‐Dias et al. 2014).

The r*Mr*B1 protein also induced H_2_O_2_ accumulation compared with the control treatments. Because H_2_O_2_ is an early marker associated with cell death and defence activation, these data suggest that oxidative stress may contribute to the necrotic lesions observed in cacao leaves (Van Breusegem and Dat 2006). Similar associations between effector‐induced cell death and ROS accumulation have been reported for BcCrh1 from *Botrytis cinerea* and RsSCR10 from *Rhizoctonia solani* (Bi et al. 2021; Niu et al. 2021).

The sequence homology between *Mr*B1 and BASIDIN, combined with their shared ability to induce ROS accumulation and cell death, supports the hypothesis that conserved effectors may retain related biological functions across pathogens (Karki et al. 2021; Newman et al. 2023). Similar functional conservation has been documented for CEC3 homologues from four *Colletotrichum* species, which induce cell death and nuclear expansion (Tsushima et al. 2021). These findings suggest that *Mr*B1 and BASIDIN may share related effector‐associated mechanisms, potentially contributing to the adaptation of *M. roreri* and *M. perniciosa* , respectively.

Conservation of three‐dimensional architecture despite primary sequence divergence is a common feature of fungal effectors and may contribute to the maintenance of biological function under evolutionary pressure (Lahfa et al. 2024). In silico analyses linked *Mr*B1 to the necrosis‐inducing secreted protein 1 (NIS1) effector family through structural similarity to MoNIS1, which adopts an eight‐stranded β‐barrel fold, a folding mode not previously described in other effectors (Han et al. 2024). NIS1 proteins are secreted fungal effectors involved in modulating plant immunity. Although MoNIS1 acts as an immune‐suppressive effector, CoNIS1 from *Colletotrichum orbiculare* and VmNIS1 from *Valsa mali* induce necrosis in *N. benthamiana*, while recombinant VmNIS1 triggers ROS accumulation (Irieda et al. 2019; Nie et al. 2022). Similarly, FsNis1 from *Fusarium sacchari* induces PAMP‐triggered immune responses in *N. benthamiana*, including ROS production and necrotic lesions (Di et al. 2024). Therefore, the necrosis and oxidative stress induced by *Mr*B1 are consistent with the functional diversity observed among NIS1 effectors.

### 3.4 rMrB1 Induces Symptoms Depending on the *T. cacao* Genotype

FPR remains a major challenge to cacao production, with only a limited number of resistant genotypes identified, most of which were developed at the Tropical Agricultural Research and Higher Education Center (CATIE) in Costa Rica. Genotypes such as CATIE‐R1, CATIE‐R4, CATIE‐R6, CC‐137, ICS‐95 and PMCT‐58 were selected to improve both yield and disease resistance (Phillips‐Mora et al. 2013). Despite being resistant, these plants are not completely immune to *M. roreri* and may exhibit mild infections with reduced sporulation (Bailey et al. 2014).

To expand our understanding of the protein's action, we evaluated its effect on contrasting cacao genotypes for resistance to FPR and WBD. After protein treatment (5 μM), distinct responses were observed among the six genotypes. The ICS‐95 genotype is widely recognised as resistant to FPR (Bekele et al. 2024) and has shown a broad spectrum of resistance to multiple isolates. In this study, ICS‐95 did not exhibit symptoms following treatment with the protein, suggesting that this genotype has the capacity to neutralise or inhibit the action of the protein compared with the other genotypes analysed.

On the other hand, the CATIE‐R4 and CATIE‐R6 genotypes inherited their resistance to FPR from UF‐273 (Dessauw et al. 2024). Although UF‐273 is genetically resistant to *M. roreri*, its interaction with the protein still allows the expression of mild symptoms. It is noteworthy that genotypes considered resistant to the disease were not completely symptom‐free, as previously reported by Bailey et al. (2014).

The strong similarity between the *Mr*B1 and BASIDIN sequences of *M. perniciosa* suggests that the action mechanisms of these proteins may be shared between the two fungal diseases. In this context, we included genotypes susceptible to WBD, such as Catongo, as well as resistant or moderately resistant genotypes, such as TSH‐1188, SCA‐6 and CCN‐51 (Faleiro et al. 2006; Silva et al. 2010; Boza et al. 2014; Royaert et al. 2016; Marssaro et al. 2020).

In our tests, the TSH‐1188 genotype showed mild symptoms on the leaf edges after treatment with the protein, while Scavina‐6 did not exhibit any symptoms. In turn, CCN‐51, known to have moderate resistance to WBD (Boza et al. 2014), had areas of necrotic lesions.

The Catongo genotype is classified as susceptible to WBD and had previously been tested in this study at a concentration of 12.5 μM. Subsequently, we used a lower concentration (5 μM) of the protein for comparison with the other genotypes at the same dosage. The Catongo leaves had larger areas of necrotic lesions, with more pronounced damage compared to the other genotypes tested. It is important to highlight that this genotype is known to express reduced levels of proteins associated with defence mechanisms against *M. perniciosa* , indicating a weaker immune response to infection (Dos Santos et al. 2020).

Similar genotype‐dependent responses have been described for the effectors ToxA and ToxB from *Pyrenophora tritici‐repentis*, which induce different levels of necrosis and chlorosis depending on the wheat genotype tested (Moffat et al. 2014; See et al. 2018, 2019). Plant sensitivity to ToxA was associated with disease severity, and removal of the susceptibility gene (*Tsn1*) increased host resistance. Furthermore, transformant strains, in which the *ToxA* gene was deleted, failed to induce necrosis in wheat genotypes susceptible to ToxA. Therefore, it is possible to gradually eliminate cultivars with the susceptibility gene by screening populations that do not show symptoms after interaction with ToxA.

In this study, different *T. cacao* genotypes showed varying levels of resistance or susceptibility to r*Mr*B1. These observations suggest that effectors, such as *Mr*B1, may allow the rapid evaluation of the response of different genotypes to the pathogen's protein without the need for prolonged testing. Genotypes such as ICS‐95, previously described as resistant to *M. roreri*, did not show symptoms after protein application. In contrast, susceptible genotypes such as Catongo showed more intense symptoms, suggesting that *Mr*B1 may be further evaluated as a candidate tool for screening susceptibility in progenies resulting from crosses aimed at pyramiding resistance genes in breeding programmes.

This study contributes to expanding knowledge about the mechanisms of action of *M. roreri* effectors. In silico structural analysis indicated similarity between *Mr*B1 and NIS1‐family effectors. The effector protein *Mr*B1 induces necrosis, membrane damage and H_2_O_2_ accumulation in plant cells. Proteomic analyses indicate that this cytotoxic response is associated with reduced abundance of proteins related to energy metabolism and proteostasis, including reduced BiP accumulation. These results indicate that this protein affects cellular homeostasis, although its precise molecular targets remain to be determined. Future studies should evaluate UPR gene expression, protein degradation inhibitor assays, co‐immunoprecipitation assays, and mitochondrial assays to further clarify the cellular pathways affected by this effector. In addition, *Mr*B1 should be tested as a susceptibility marker for early screening of young cacao plants. This approach may reduce time and costs in breeding programmes, allow genotype evaluation in regions free of FPR, and facilitate the identification of promising plant materials. The thermostability of r*Mr*B1 further supports its potential use in future biotechnological applications.

## 4 Experimental Procedures

### 4.1 Selection of the Effector Candidate

The sequence of BASIDIN, an effector protein previously characterised in *M. perniciosa* , was used in BLASTp (http://blast.ncbi.nlm.nih.gov/Blast.cgi) to search for homologues in other plant‐pathogenic fungal species. A hypothetical protein from *M. roreri* with 92% sequence identity to BASIDIN was selected for this study and called *Mr*B1.

### 4.2 In Silico Analyses

To characterise the *Mr*B1 protein, several bioinformatics tools were used. Initially, SignalP 6.0 (https://services.healthtech.dtu.dk/service.php?SignalP‐6.0) was used to predict the presence of a secretion signal peptide, while TMHMM 2.0 (http://www.cbs.dtu.dk/services/TMHMM‐2.0/) was used to predict transmembrane helices. The subcellular location of the protein was evaluated using the DeepLoc‐2.0 program (https://services.healthtech.dtu.dk/service.php?DeepLoc‐2.0). The secondary structure was provided by the PSIPRED 4.0 server (http://bioinf.cs.ucl.ac.uk/psipred/), and conserved domain analysis was performed using InterProScan (https://www.ebi.ac.uk/interpro/search/sequence/). The physicochemical properties of the protein's primary structure were predicted using the Expasy ProtParam tool (https://web.expasy.org/protparam/). Intrinsically disordered regions were predicted using RFPR‐IDP (http://bliulab.net/RFPR‐IDP/server). Additionally, to predict effector characteristics, we used EffectorP 3.0 (Sperschneider and Dodds 2022) based on the amino acid sequence.

The three‐dimensional structure of mature *Mr*B1 was predicted using the AlphaFold2 algorithm through ColabFold v1.6.1 (https://colab.research.google.com/github/sokrypton/ColabFold/). Multiple sequence alignments were generated using MMseqs2, and the final model was selected based on the highest predicted local distance difference test (pLDDT) score. Structural homologue searches were performed using the DALI server (http://ekhidna2.biocenter.helsinki.fi/dali/), comparing the predicted protein topology against structures deposited in the Protein Data Bank (PDB). Structural visualisation and figure preparation were performed using Mol* Viewer, a free, open‐source, web‐based molecular visualisation toolkit (https://molstar.org/).

### 4.3 Transformation, Expression and Purification of Recombinant *Mr*B1 in *E. coli*

The pET28a(+) vector containing the synthetic ORF of *Mr*B1 at the NdeI and XhoI restriction sites (BIOMATIK) was used for transformation and expression in *E. coli* . After insertion of the recombinant plasmid into the Rosetta (DE3) strain by heat shock, the cells were inoculated on Luria Bertani (LB) plates containing 50 μg mL^−1^ kanamycin and 34 μg mL^−1^ chloramphenicol and incubated at 37°C. The transformed cells were initially cultured in 5 mL of LB medium and subsequently in 400 mL of the same medium, supplemented with kanamycin and chloramphenicol. Incubation was carried out at 37°C, with stirring at 180 rpm, reaching an optical density of 0.8 (OD_600 nm_). The expression of the r*Mr*B1 protein (recombinant *Mr*B1) was induced with IPTG (0.4 mM), followed by incubation at 37°C with agitation at 180 rpm for 4 h.

After induction, cells were collected by centrifugation (10,000 rpm, 4°C, 10 min). The cell pellet was resuspended in lysis buffer (Tris–HCl pH 7.4, containing 100 mg mL ^−1^ lysozyme) and incubated at room temperature for 30 min. The cell suspension was then sonicated (8 s on/20 s off, amplitude of 70%) and centrifuged at 14,000 rpm, 4°C, for 20 min. The pellet (insoluble fraction) and supernatant (soluble fraction) were collected for purification by affinity chromatography using the HisTALON kit (TaKaRa), according to the manufacturer's instructions. The expression, purity and homogeneity of the fractions were evaluated by SDS‐PAGE at 12.5% and stained with 0.08% colloidal Coomassie blue (Neuhoff et al. 1988). The protein was dialysed against 10 mM Tris–HCl buffer, and its concentration was determined using the Qubit Protein Assay kit, following the manufacturer's protocol (Invitrogen).

### 4.4 Confirmation of Expression by Mass Spectrometry

To confirm the expression and identity of the recombinant *Mr*B1 protein, the samples were initially subjected to SDS‐PAGE separation. The band corresponding to the expected molecular weight (~20 kDa) was carefully excised from the gel and subjected to dye removal with acetonitrile. The fragment was then enzymatically digested with trypsin at 37°C for 16 h. The resulting peptides were analysed by mass spectrometry using the 6545 Q‐TOF system coupled to the LC 1290 Infinity II solution (Agilent Technologies).

### 4.5 CD Spectroscopy

CD spectra of r*Mr*B1 were obtained using a Jasco J‐815 spectropolarimeter. Before data collection, samples were dialysed in 5 mM Tris–HCl. Data acquisition occurred in the wavelength range of 190 to 240 nm, with 1.0 nm intervals, at temperatures of 25°C and 95°C, using a 1.0 mm cuvette. Each spectrum corresponds to the average of six consecutive scans. For secondary structure analysis, the CD spectra were processed using the online platform available at: https://capito.bellstedt‐lab.ch/.

### 4.6 Evaluation of the Effect of r*Mr*B1 in Transgenic *N. benthamiana* Cells

The suspension culture of transgenic *N. benthamiana* cells (16C, GFP strain) was performed as described by Do Carmo Santos et al. (2024).

### 4.7 Action of r*Mr*B1 in *N. benthamiana* Cells

The activity of r*Mr*B1 was evaluated in *N. benthamiana* cell suspensions expressing GFP. For this, 300 μL aliquots of exponentially growing cells were centrifuged at 3000 rpm for 30 s. The culture medium was removed and the cells were treated with: (I) Control (Tris–HCl 10 mM, pH 7.4); (II) 2.5 μM r*Mr*B1; (III) 5 μM r*Mr*B1; (IV) 5 μM r*Mp*NEP2. Untreated cells were used for viability control. All treatments were completed with a volume of 300 μL. After treatment for 1 h, 100 μL of each cell culture was applied to each slide and examined using an Olympus BX51 epifluorescence microscope, with an excitation filter at 488 nm and an emission filter at 510 nm. Images were acquired and analysed using Image Pro Plus v. 4.1 (Media Cybernetics). The analysis focused on GFP expression for observation of morphological changes, which were compared with those of the control group. Quantitative measurements were performed to provide a detailed assessment of the treatment effects on transgenic cells. Cell counting was performed using ImageJ2 (v. 2.9.0/1.53t) (Rueden et al. 2017). The comparison between the percentages of cells after treatments was made using ANOVA, followed by the Tukey test (*p* < 0.05).

### 4.8 Cell Membrane Permeability

The effect of r*Mr*B1 on the membrane permeability of exponentially staged *N. benthamiana* cells expressing GFP was evaluated using PI staining. The culture medium was removed and the cells were treated with: (I) 10 mM Tris–HCl, pH 7.4; (II) 2.5 μM r*Mr*B1; (III) 5 μM r*Mr*B1; (IV) 10 μM r*Mr*B1. After treatment, the cells were incubated with 20 μg mL^−1^ PI for 15 min and observed by epifluorescence microscopy (Olympus BX51), using an excitation filter at 535 nm and an emission filter at 617 nm. The presence of PI staining in the nucleus was interpreted as damage to the cell membrane. The quantification of cells with membrane damage (nonviable cells) and cells expressing GFP (viable cells) was calculated and analysed as detailed in Section 4.6.

### 4.9 Electrolyte Leakage

*Nicotiana benthamiana* cell suspensions were treated with r*Mr*B1 at a concentration of 2.5 μM and with 10 mM Tris–HCl buffer (control). Each sample was adjusted to a final volume of 15 mL and incubated under stirring at 100 rpm at 25°C for 24 h. After this period, the electrical conductivity (EC) of the supernatant was determined using a portable digital conductivity meter (CD‐4322). The conductivity value of the cell‐free MS medium (EC_medium_ = mS cm^−1^) was used for background correction. The total conductivity (EC_total_ = mS cm^−1^) was obtained after boiling the samples for 15 min. The percentage of spillage was calculated using the equation: Leakage (%) = [(EC − EC_medium)/(EC_total − EC_medium)] × 100.

### 4.10 Proteomic Analysis

#### 4.10.1 Protein Extraction From *N. benthamiana* Cells

Total proteins were extracted from plant cells treated with 5 μM r*Mr*B1 for 1 h. The control group consisted of cells treated with 10 mM Tris–HCl buffer. Extraction was performed from a biological pool, formed by three independent cell samples subjected to the same treatment. After treatment, the samples were centrifuged, the supernatant was discarded, and the pellet was rapidly frozen in liquid nitrogen and lyophilised. Approximately 0.07 g of the dried material was macerated in liquid nitrogen in the presence of polyvinylpyrrolidone (PVPP) and resuspended in ice‐cold acetone. The homogenate was centrifuged (14,000 rpm, 5 min, 4°C) and the procedure was repeated twice. The pellet was air‐dried, washed three times with 10% trichloroacetic acid (TCA) in acetone, and centrifuged under the same conditions. Next, the pellet was sonicated (6 pulses of 5 s on/10 s off) and precipitated in 10% trichloroacetic acid (TCA) in water for 1 h at −20°C. After further centrifugation, the supernatant was discarded and the pellet washed three times with 80% acetone. The final material was resuspended in 200 μL of 8 M urea. Protein concentration was determined using the 2D Quant kit (GE Healthcare Life Sciences). For quality control, 30 μg of each sample was analysed by SDS‐PAGE.

#### 4.10.2 Tryptic Digestion

After extraction, the samples were subjected to tryptic digestion following the protocol described by Villén and Gygi (2008), with minor modifications. Aliquots containing 100 μg of total protein were diluted 1:1 in water, reduced with dithiothreitol (DTT), and subsequently alkylated with iodoacetamide (IAA). The sample volume was then adjusted with a 50 mM ammonium bicarbonate solution containing CaCl_2_ in a 1:5 ratio and incubated with trypsin at 37°C for 16 h. Digestion was stopped by the addition of trifluoroacetic acid (TFA) until pH < 2. The peptides were desalted using C18 tips (Thermo Fisher) and eluted with 50 μL of a solution containing 50% acetonitrile and 0.1% formic acid.

#### 4.10.3 LC–MS/MS Analysis

The peptides were analysed using an Agilent 1290 Infinity II liquid chromatography system coupled to an Agilent 6545 LC/QTOF mass spectrometer. Each sample was injected in technical triplicate and separated on a reversed‐phase C18 column (AdvanceBio Peptide Mapping, 2.1 × 250 mm; Agilent), maintained at 55°C. Separation was performed using a 20‐min gradient using mobile phases A (water with 0.1% formic acid) and B (acetonitrile with 0.1% formic acid). The percentages of phase B were: 5%–35% (1–10 min), 35%–70% (11–14 min), 70%–100% (16–18 min), and 100% (18–20 min), followed by 5 min of re‐equilibration at 5%. Ionisation was performed by electrospray in AutoMS/MS mode, by selecting up to 10 precursors per cycle (threshold 1000 counts; minimum purity 30%; charges 2+, 3+, > 3+ or unknown). Collision energy followed the equation (slope × *m*/*z*)/100 + offset (slope 3.1–5; offset −4.8 to 10). The instrument parameters were gas flow rate 13 L min^−1^, 325°C; skimmer 56 V; capillary 4000 V. Instrument control was performed using the Agilent MassHunter Acquisition software.

#### 4.10.4 Data Identification and Processing

Spectra were acquired in technical triplicate and processed on a Spectrum Mill (Rev BI.07.08.214), using a noise threshold of 10 counts, an MH^+^ window of 200–6000 Da, a retention tolerance of ±60 s, and an *m*/*z* tolerance of ±1.4. Although the proteins were extracted from *N. benthamiana*, searches were performed against the *Nicotiana tabacum* proteome (NCBI, Dec/2025) due to its more complete and curated annotation. Search parameters included up to four missing cleavages, carbamidomethylation (C) as a fixed modification, and the following variables: deamidation (N); phosphorylation (S); oxidation (M); pyroglutamic acid (N‐terminal) (Q); with modifications to threonine (T) and tyrosine (Y). Precursor tolerance was set at ±20 ppm and the minimum combined intensity at 10%. The validation used a false positive rate (FDR) < 1% at the peptide and protein levels, retaining only proteins containing peptides with a score > 5 and an SPI > 60%.

Protein abundance was estimated by summing the intensities of the peptides assigned to each protein. The fold change (FC) was calculated as the ratio of the mean normalised intensities between the control and r*Mr*B1 conditions. Statistical significance was assessed using an unpaired *t*‐test. Proteins with |FC| > 1.5 were considered to have a marked variation in relative abundance between conditions.

The heatmap was constructed to visualise global protein abundance patterns across experimental conditions, based on intensity values transformed into log_2_(*x* + 1). Hierarchical clustering was performed using Euclidean distance and the complete linkage method. Functional annotation of the proteins was obtained using the PANZER2 pipeline (http://ekhidna2.biocenter.helsinki.fi/sanspanz/), which generated Gene Ontology (GO) terms for Biological Process, Molecular Function, and Cellular Component. Statistical and visualisation analyses were conducted in the R environment (v. 4.5.1).

### 4.11 PPI Network

The PPI network was constructed from proteins with altered abundance: up‐accumulated (proteins exclusive to the r*Mr*B1 treatment) and down‐accumulated (proteins exclusive to the control and common proteins that showed reduced abundance in the r*Mr*B1 treatment). Each selected protein was analysed in the STRING v. 11.5 database (https://string‐db.org/), configured to include all active interaction sources (text mining, experiments, databases, co‐expression, neighbourhood, gene fusion, and co‐occurrence), adopting a small high‐confidence score (0.700) and allowing up to 50 interactors for the 1st and 2nd layers. The results were exported in TSV format and integrated into Cytoscape v. 3.9.1 (http://www.cytoscape.org/) to construct the consolidated network, whose topological properties (centrality and modularity) were calculated using the iGraph package in R (v. 4.5.1). For each cluster identified in the topological analysis, a functional enrichment analysis was performed directly in STRING, using the complete *N. tabacum* genome as statistical background, with correction for multiple tests using the Benjamini–Hochberg procedure.

### 4.12 Western Blot

To validate the data obtained by mass spectrometry, an immunodetection assay was performed to evaluate the presence and accumulation of BiP. For this, 30 μg of total proteins from *N. benthamiana* cells treated with r*Mr*B1 and from the control group were separated by 12.5% SDS‐PAGE using a prestained molecular weight marker (Kaleidoscope, Bio‐Rad). A mirror gel was prepared in parallel to confirm the banding pattern before transfer. The proteins were transferred to a nitrocellulose membrane (Bio‐Rad) in transfer buffer (25 mM Tris, 0.2 M glycine, 10% methanol) for 1 h and 30 min at 250 mA. The membranes were blocked overnight in Tris‐buffered saline with Tween 20 (TBS‐T) containing 10% skim milk, incubated with anti‐BiP primary antibodies for 1 h under agitation, washed with TBS‐T, and subsequently incubated with alkaline phosphatase‐conjugated anti‐rabbit secondary antibodies for 1 h at room temperature. Detection was performed using 5‐bromo‐4‐chloro‐3‐indolyl phosphate/nitrotetrazolium blue (BCIP/NBT) substrate in 1 M Tris–HCl buffer pH 9.5 until band visualisation. BiP protein accumulation was quantified from the membrane imaging using the ImageJ2 software (v. 2.9.0/1.53t).

### 4.13 Functional Assay in *T. cacao* Plants

Eight‐week‐old cacao plants were selected for functional assays. The plants were produced from open‐pollinated seeds of the clones ICS‐95, UF‐273, Scavina‐6, TSH‐1188, CCN‐51 and Catongo, sown in tubes at the Cocoa Research Center. Subsequently, the seedlings were transferred to facilities at State University of Santa Cruz (UESC) in Ilhéus, Bahia, Brazil, where they were maintained under controlled temperature and humidity conditions. After removing 1/3 of the leaf from the apex, the remaining 2/3 of the leaf was sprayed with 1 mL of r*Mr*B1 at concentrations of 5 μM and 12.5 μM. In addition, 0.5% urea was used as an adjuvant to improve protein absorption by the leaves. The control group received treatment with spraying of 10 mM Tris–HCl buffer with and without urea. The treated leaves were monitored and photographed until the 10th day. The images were uploaded to ImageJ2 (v. 2.9.0/1.53t), where the total leaf area and the lesion area were identified and measured. Quantification was performed using the formula: lesion area (%) = (lesion area/total leaf area) × 100, to calculate the percentage of the lesioned area in relation to the total leaf area.

Additionally, 5 μM r*Mr*B1 was injected into cacao fruits of the Parazinho genotype (common cacao), maintained in a cabruca system on the UESC premises. For each treatment, approximately five applications were performed in three distinct regions of the fruits. Five days after application, the fruits were photographed and evaluated for the presence of symptoms.

### 4.14 Detection of H_2_O_2_

The presence of H_2_O_2_ was evaluated using the DAB staining method (Thordal‐Christensen et al. 1997). After treating cacao leaves with 5 μM r*Mr*B1, 1 cm diameter discs were cut from the treated leaves after 24 h. The discs were immersed in a solution of 1 mg mL^−1^ DAB dissolved in distilled water acidified with HCl (pH 3.8). Subsequently, they were vacuum‐sealed for 30 min and then incubated in the same solution for 24 h at room temperature in the dark. To remove DAB and chlorophyll residues, the leaf tissues were boiled in 96% and 50% ethanol, respectively, for 30 min. The results were observed and recorded using a stereomicroscope. Images of the leaf discs were loaded into a computer running the ImageJ2 software (v. 2.9.0/1.53t). With the aid of image analysis tools, the total area of the leaf disc and the area with DAB accumulation were identified and measured.

## Author Contributions

**Keilane Silva Farias:** methodology, investigation. **Taís Araújo Santos:** conceptualization, methodology, formal analysis, writing – original draft, investigation, validation. **Carlos Henrique de Carvalho Neto:** methodology. **Uilson Vanderlei Lopes:** methodology, writing – review and editing. **Monaliza Macêdo Ferreira:** methodology, investigation, formal analysis, data curation, software. **Maria Luíza do Carmo Santos:** methodology, writing – review and editing, visualization. **Ronan Xavier Corrêa:** writing – review and editing. **Carlos Priminho Pirovani:** conceptualization, funding acquisition, project administration, supervision, resources.

## Funding

This work was supported by Conselho Nacional de Desenvolvimento Científico e Tecnológico (309466/2023‐7, 403680/2024‐7 and 421787/2021‐0), Coordenação de Aperfeiçoamento de Pessoal de Nível Superior (0001), Fundação de Amparo à Pesquisa do Estado da Bahia (BOLO583/2022).

## Conflicts of Interest

The authors declare no conflicts of interest.

## Supporting information


**Figure S1:** Prediction of secondary structure and intrinsically disordered regions (IDRs) in *Mr*B1, based on the amino acid sequence without the signal peptide. (A) The secondary structure was predicted using the PSIPRED 4.0 server, which estimated the following percentages: α‐helix 2.07%, β‐sheet 32.41%, and irregular regions (coil) 65.52%. (B) The RFPR‐IDP server predicted two intrinsically disordered regions in *Mr*B1.

**Figure S2:** Mass spectrometry analysis of the r*Mr*B1 protein. (A) Mass spectrum showing the peaks corresponding to the peptide ions detected from the recombinant protein, expressed in a heterologous system and excised from SDS‐PAGE gel for mass spectrometry analysis. Each coloured dot represents an identified peptide, with the *x*‐axis indicating the mass‐to‐charge ratio (*m*/*z*) and the *y*‐axis showing the relative signal intensity (%). The labels indicate the peptide sequences and their respective charge states. (B) Linear peptide coverage map showing the amino acid sequence regions detected by mass spectrometry. The analysis was performed using the mature protein sequence, excluding the predicted signal peptide, and revealed total coverage of 37.2% of the sequence. (C) Table listing the identified peptides with their positions in the protein, *m*/*z* values, charge states, and relative intensities.

**Figure S3:** Epifluorescence microscopy images of *Nicotiana benthamiana* cells expressing GFP. Untreated cells have intact cellular integrity. Cells treated with 2.5 μM r*Mr*B1 show damage to cellular integrity, suggesting increased membrane permeability, GFP loss, and collapse 1 h after treatment.

**Figure S4:** Protein profile of *Nicotiana benthamiana* cells. Total proteins were extracted from plant cells treated with 5 μM r*Mr*B1 for 1 h, while the control group received only 10 mM Tris–HCl buffer. For quality control analysis, 30 μg of each sample was loaded onto an SDS‐PAGE gel. The molecular weight marker (kDa) is indicated on the left.

**Figure S5:** Induction of necrotic lesions in *Theobroma cacao* fruit. Fruit of the Parazinho genotype five days after injection of r*Mr*B1 (5 μM) and Tris–HCl buffer (control). The regions treated with r*Mr*B1 show evident necrotic spots. Each treatment was applied approximately five times to three distinct regions of the fruit.

**Table S1:** Quantification of cell death in *Nicotiana benthamiana* cell suspensions based on fluorescence.

**Table S2:** Quality‐filtered proteins (FDR < 1%, score > 5, SPI > 60%) identified and quantified by LC–MS/MS from *Nicotiana benthamiana* cells under control and r*Mr*B1 treatment.

**Table S3:** Quantification of leaf lesion and hydrogen peroxide in *Theobroma cacao* .

## Data Availability

The data that support the findings of this study are available in the Supporting Information of this article.
