# Supplementary figures and images for: The Moniliophthora roreri Effector Mr B1 Affects Cellular Homeostasis and Induces Genotype‐Dependent Necrosis in Theobroma cacao
Source: Mol Plant Pathol. 2026 Jul 12;27(7):e70316. doi: 10.1111/mpp.70316 (PMC13358014; doi:10.1111/mpp.70316)

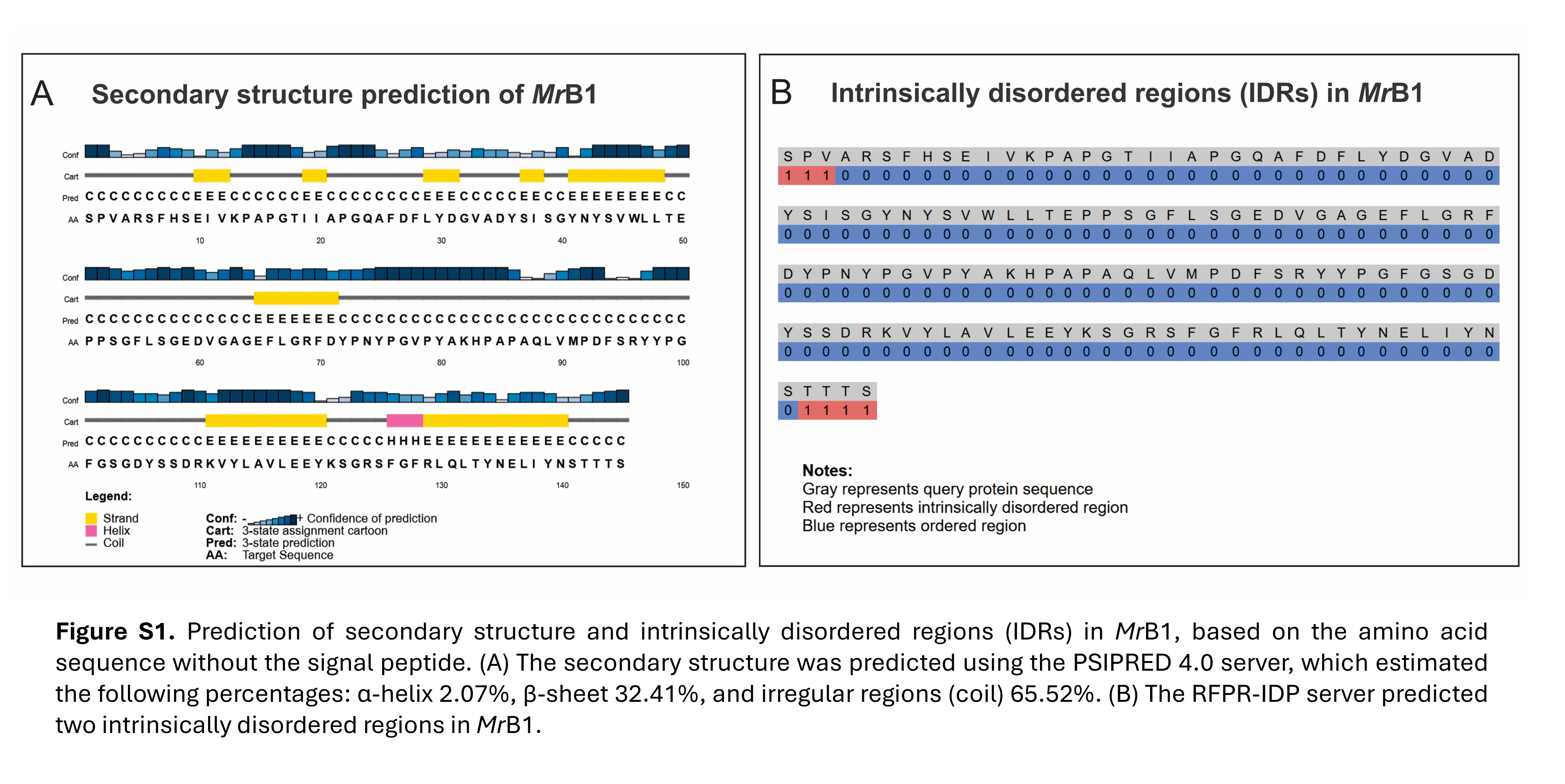

Supplement: Supplementary file 1 — Figure S1: Prediction of secondary structure and intrinsically disordered regions (IDRs) in MrB1, based on the amino acid sequence without the signal peptide. (A) The secondary structure was predicted using the PSIPRED 4.0 server, which estimated the following percentages: α‐helix 2.07%, β‐sheet 32.41%, and irregular regions (coil) 65.52%. (B) The RFPR‐IDP server predicted two intrinsically disordered regions in MrB1. [file MPP-27-e70316-s003.png]

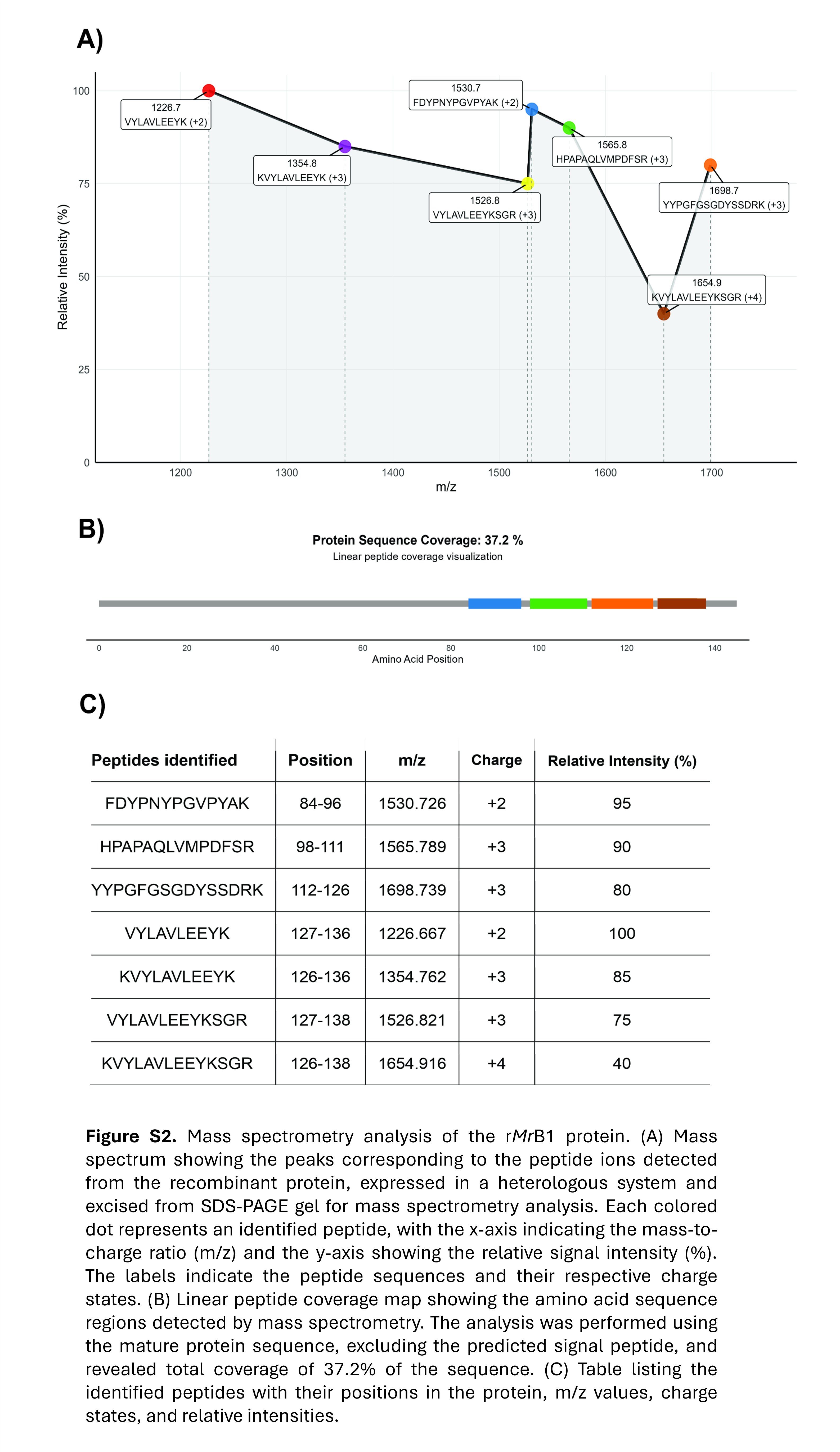

Supplement: Supplementary file 2 — Figure S2: Mass spectrometry analysis of the rMrB1 protein. (A) Mass spectrum showing the peaks corresponding to the peptide ions detected from the recombinant protein, expressed in a heterologous system and excised from SDS‐PAGE gel for mass spectrometry analysis. Each coloured dot represents an identified peptide, with the x‐axis indicating the mass‐to‐charge ratio (m/z) and the y‐axis showing the relative signal intensity (%). The labels indicate the peptide sequences and their respective charge states. (B) Linear peptide coverage map showing the amino acid sequence regions detected by mass spectrometry. The analysis was performed using the mature protein sequence, excluding the predicted signal peptide, and revealed total coverage of 37.2% of the sequence. (C) Table listing the identified peptides with their positions in the protein, m/z values, charge states, and relative intensities. [file MPP-27-e70316-s002.png]

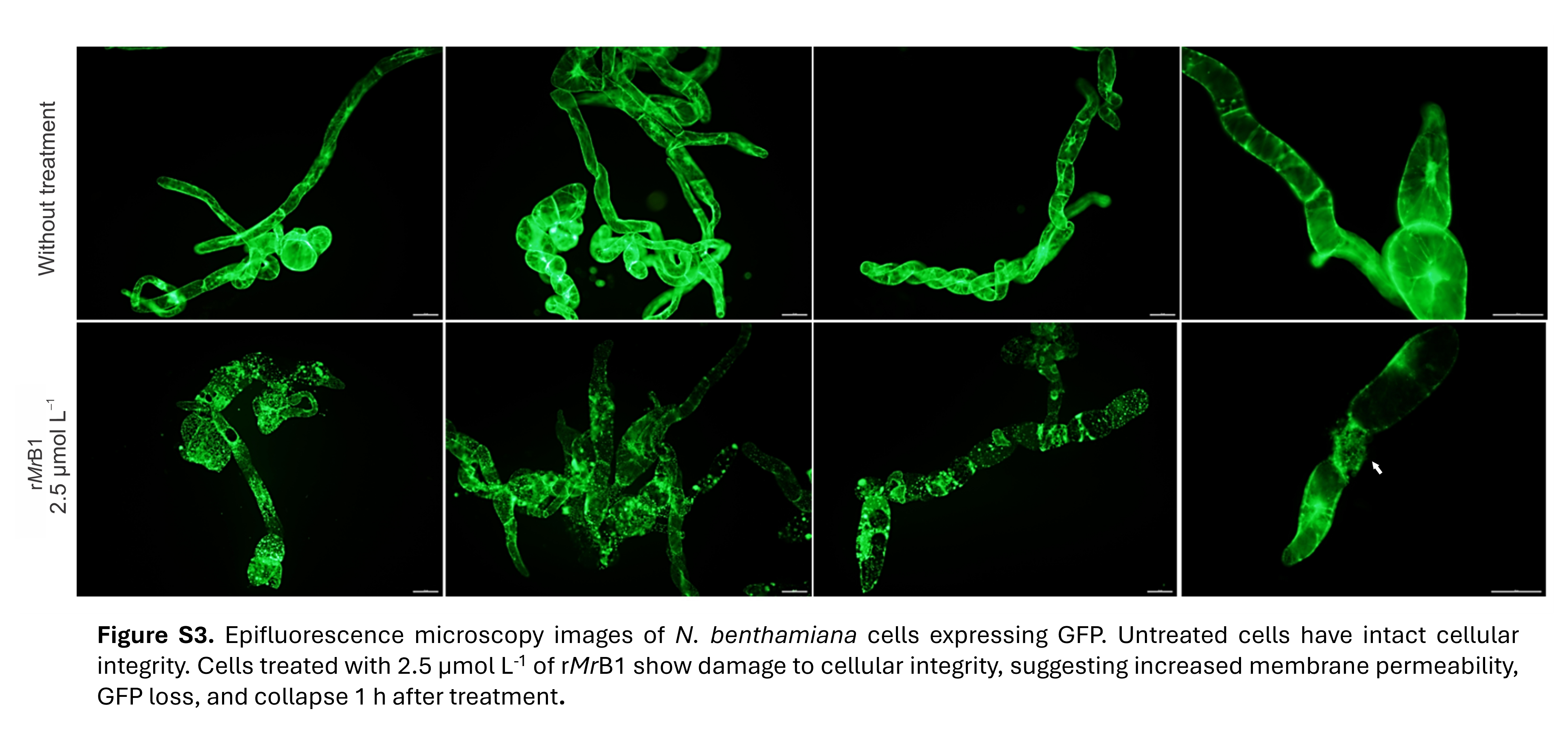

Supplement: Supplementary file 3 — Figure S3: Epifluorescence microscopy images of Nicotiana benthamiana cells expressing GFP. Untreated cells have intact cellular integrity. Cells treated with 2.5 μM rMrB1 show damage to cellular integrity, suggesting increased membrane permeability, GFP loss, and collapse 1 h after treatment. [file MPP-27-e70316-s007.png]

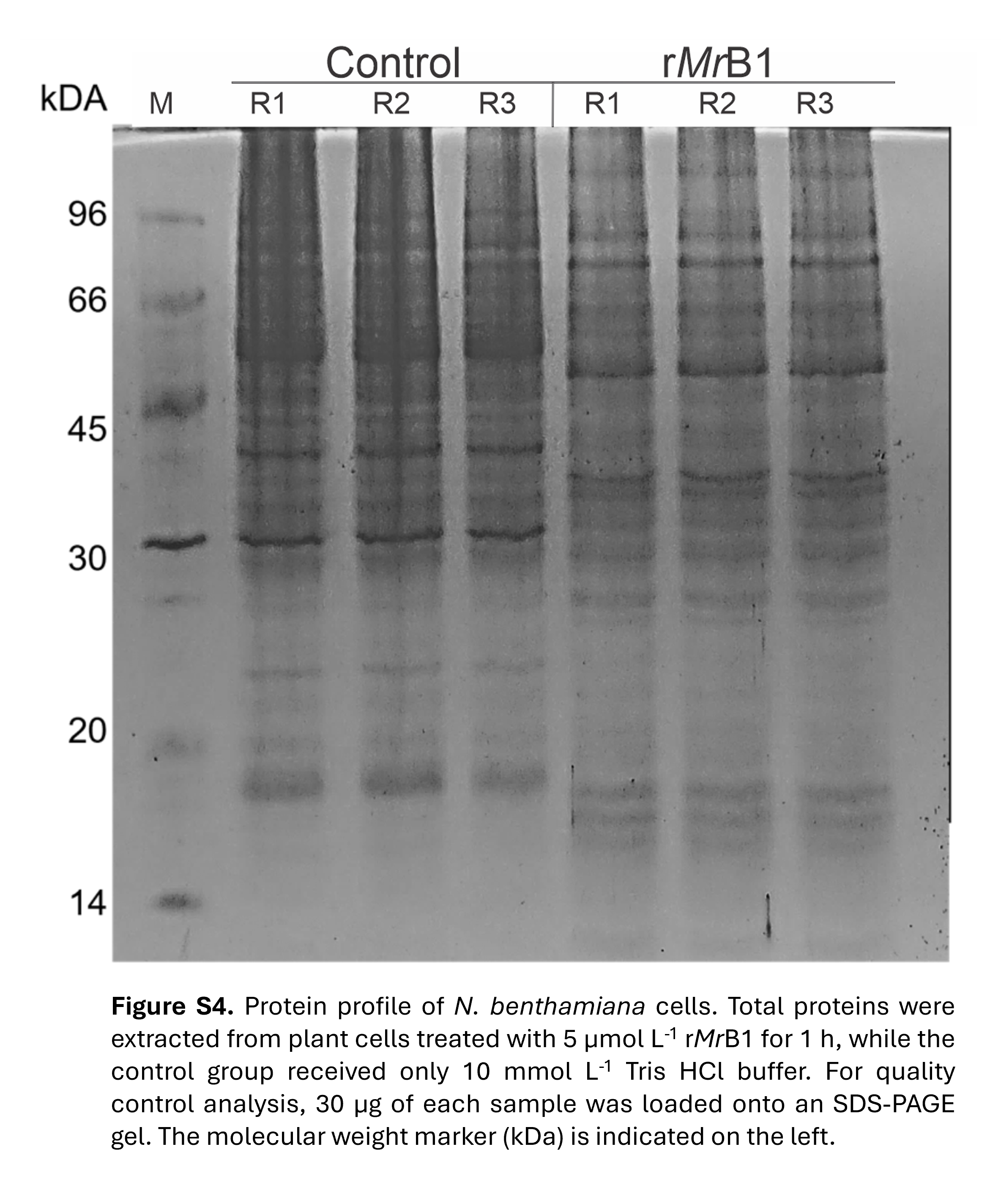

Supplement: Supplementary file 4 — Figure S4: Protein profile of Nicotiana benthamiana cells. Total proteins were extracted from plant cells treated with 5 μM rMrB1 for 1 h, while the control group received only 10 mM Tris–HCl buffer. For quality control analysis, 30 μg of each sample was loaded onto an SDS‐PAGE gel. The molecular weight marker (kDa) is indicated on the left. [file MPP-27-e70316-s001.png]

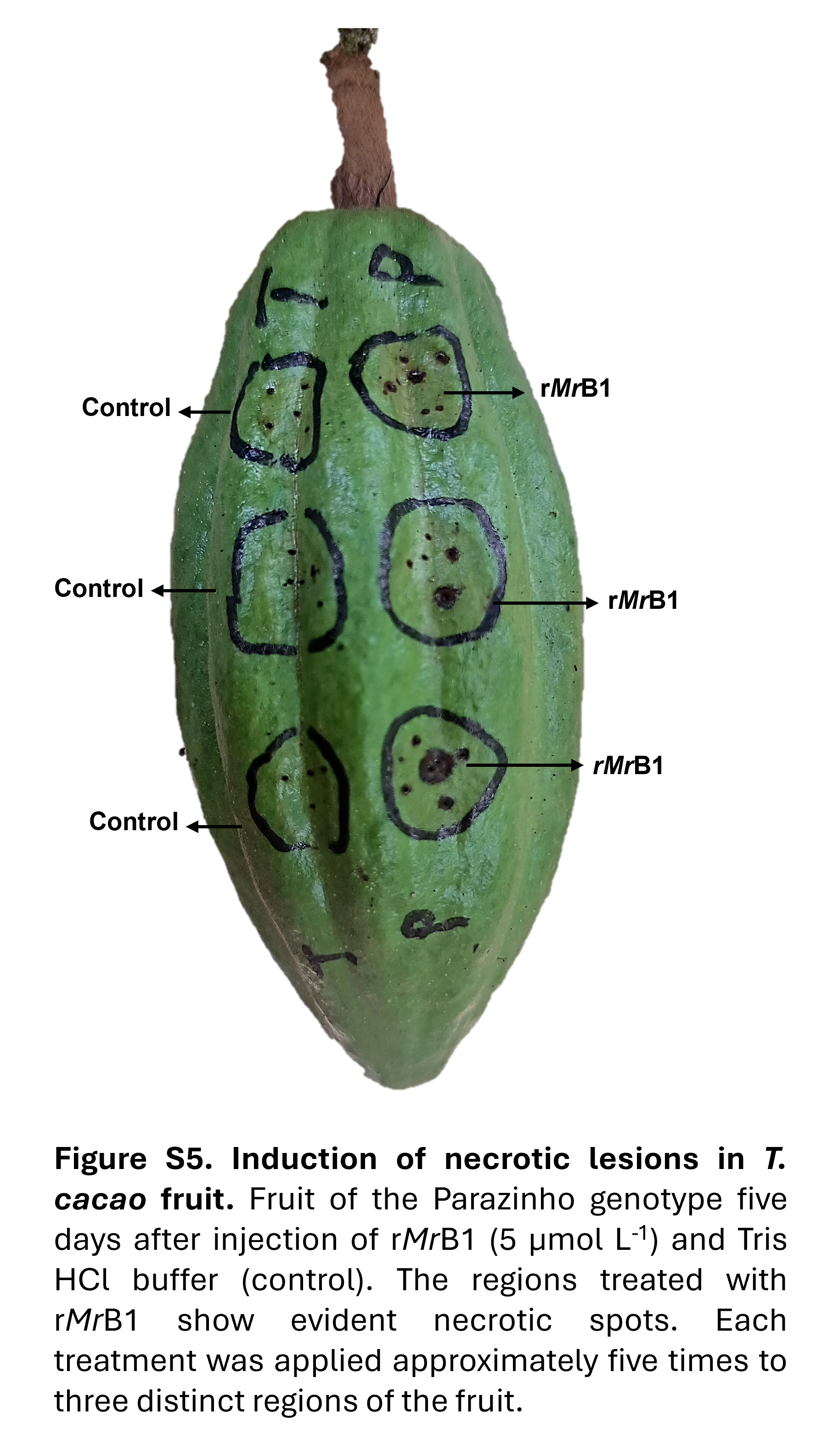

Supplement: Supplementary file 5 — Figure S5: Induction of necrotic lesions in Theobroma cacao fruit. Fruit of the Parazinho genotype five days after injection of rMrB1 (5 μM) and Tris–HCl buffer (control). The regions treated with rMrB1 show evident necrotic spots. Each treatment was applied approximately five times to three distinct regions of the fruit. [file MPP-27-e70316-s008.png]
